# miR‐424‐5p reduces ribosomal RNA and protein synthesis in muscle wasting

**DOI:** 10.1002/jcsm.12266

**Published:** 2017-12-07

**Authors:** Martin Connolly, Richard Paul, Roser Farre‐Garros, Samantha A. Natanek, Susannah Bloch, Jen Lee, Jose P. Lorenzo, Harnish Patel, Cyrus Cooper, Avan A. Sayer, Stephen J. Wort, Mark Griffiths, Michael I. Polkey, Paul R. Kemp

**Affiliations:** ^1^ Molecular Medicine Section National Heart and Lung Institute, Imperial College London South Kensington Campus London SW7 2AZ UK; ^2^ National Institute for Health Research Respiratory Biomedical Research Unit Royal Brompton and Harefield NHS Foundation Trust and Imperial College London London SW3 6NP UK; ^3^ MRC Lifecourse Epidemiology Unit University of Southampton, Southampton General Hospital Southampton SO16 6YD UK; ^4^ NIHR Southampton Biomedical Research Centre University of Southampton and University Hospital Southampton NHS Foundation Trust Southampton UK; ^5^ AGE Research Group, Institute of Neuroscience and Institute for Ageing Newcastle University Newcastle upon Tyne UK; ^6^ NIHR Newcastle Biomedical Research Centre Newcastle University and Newcastle upon Tyne Hospitals NHS Foundation Trust Newcastle upon Tyne UK; ^7^ Inflammation, Repair and Development National Heart and Lung Institute, Imperial College London South Kensington Campus London SW7 2AZ UK

**Keywords:** Ribosomal RNA synthesis, microRNA, Protein synthesis, Muscle wasting

## Abstract

**Background:**

A loss of muscle mass occurs as a consequence of a range of chronic and acute diseases as well as in older age. This wasting results from an imbalance of protein synthesis and degradation with a reduction in synthesis and resistance to anabolic stimulation often reported features. Ribosomes are required for protein synthesis, so changes in the control of ribosome synthesis are potential contributors to muscle wasting. MicroRNAs (miRNAs) are known regulators of muscle phenotype and have been shown to modulate components of the protein synthetic pathway. One miRNA that is predicted to target a number of components of protein synthetic pathway is miR‐424‐5p, which is elevated in the quadriceps of patients with chronic obstructive pulmonary disease (COPD).

**Methods:**

Targets of miR‐424‐5p were identified by Argonaute2 pull down, and the effects of the miRNA on RNA and protein expression were determined by quantitative polymerase chain reaction and western blotting in muscle cells *in vitro*. Protein synthesis was determined by puromycin incorporation *in vitro*. The miRNA was over‐expressed in the *tibialis anterior* muscle of mice by electroporation and the effects quantified. Finally, quadriceps expression of the miRNA was determined by quantitative polymerase chain reaction in patients with COPD and intensive care unit (ICU)‐acquired weakness and in patients undergoing aortic surgery as well as in individuals from the Hertfordshire Sarcopenia Study.

**Results:**

Pull‐down assays showed that miR‐424‐5p bound to messenger RNAs encoding proteins associated with muscle protein synthesis. The most highly enriched messenger RNAs encoded proteins required for the Pol I RNA pre‐initiation complex required for ribosomal RNA (rRNA) transcription, (PolR1A and upstream binding transcription factor). *In vitro*, miR‐424‐5p reduced the expression of these RNAs, reduced rRNA levels, and inhibited protein synthesis. In mice, over‐expression of miR‐322 (rodent miR‐424 orthologue) caused fibre atrophy and reduced upstream binding transcription factor expression and rRNA levels. In humans, elevated miR‐424‐5p associated with markers of disease severity in COPD (FEV_1_%), in patients undergoing aortic surgery (LVEF%), and in patients with ICU‐acquired weakness (days in ICU). In patients undergoing aortic surgery, preoperative miR‐424‐5p expression in skeletal muscle was associated with muscle loss over the following 7 days.

**Conclusions:**

These data suggest that miR‐424‐5p regulates rRNA synthesis by inhibiting Pol I pre‐initiation complex formation. Increased miR‐424‐5p expression in patients with conditions associated with muscle wasting is likely to contribute to the inhibition of protein synthesis and loss of muscle mass.

## Introduction

Altered protein synthesis occurs in a broad range of physiological and pathological situations including development and growth, cancer, and cell hypertrophy and atrophy.^1^ Changing protein synthesis can be achieved not only by regulating the activity of the protein synthetic machinery as is well described in studies of the insulin‐like growth factor (IGF) signalling pathway^2^, ^3^ but also by altering the number of ribosomes thereby altering the capacity of the system. Indeed the IGF‐1/Akt/mammalian Target Of Rapamycin (mTOR) pathway contributes to the regulation of ribosome synthesis.^1^ To increase or reduce the ribosomal number requires a change in the balance of the synthesis and degradation of ribosomal proteins and ribosomal RNA (rRNA). rRNAs are synthesized in the nucleolus, a region of the nucleus, and increased rRNA synthesis is indicated by an increase in nucleolar size.^1^ Unlike messenger RNAs (mRNA), rRNAs are synthesized by RNA polymerase I following the formation of a pre‐initiation complex (PIC) that also requires the transcription factors upstream binding transcription factor (UBTF) and RRN3 for full activity. rRNA makes up ~80% of total cellular RNA,^4^ and mammalian ribosomes contain 80 proteins with ribosome biogenesis requiring in excess of 150 trans‐acting accessory factors and multiple small nucleolar RNAs.^1^, ^5^ Synthesizing ribosomes is therefore energetically expensive, and as a result, the rate of ribosome synthesis is tightly regulated to optimize ribosomal content for the current conditions.^6^, ^7^ Ribosome biogenesis is therefore increased in times of growth or suppressed when there is no requirement for growth or when nutrient supplies are limited or need to be diverted.^1^

In the muscle, protein turnover is important not only for maintaining the tissue itself but also to provide amino acids as a source of both carbon and energy for other tissues.^8^ For example, a number of studies have shown that muscle‐derived amino acids are used by the immune system at times of increased demand,^9^, ^10^, ^11^ and it is interesting to note that excessive training by athletes can lead to chronic immunosuppression and an increased susceptibility to respiratory infections.^12^ Similarly, the increase in muscle loss in chronic disease may reflect an increase in the requirement for repair to the damaged tissue and be promoted in part by the release of inflammatory cytokines.^13^ Consequently, changes in protein turnover in muscle occur in chronic and acute disease and in normal aging, such that muscle wasting is common in patients with a range of chronic diseases and in the intensive care unit (ICU) and in older individuals, reviewed in Wolfe.^8^ Furthermore, a number of studies of these conditions have shown that individuals become resistant to anabolic stimuli.^14^, ^15^, ^16^, ^17^ The mechanisms that underlie this anabolic resistance remain to be fully elucidated but are likely to contribute significantly to the loss of muscle mass in patients with chronic diseases and older people.^14^, ^18^ In turn, the sequelae of reduced muscle mass are impaired physical performance, associated poor quality of life, and, potentially, reduced survival.^19^, ^20^, ^21^

MicroRNAs (miRNAs) are regulators of protein expression that modulate cell phenotype by either blocking translation or promoting degradation of a set or sets of mRNAs.^22^ These small RNAs are critical regulators of cell phenotype both under normal physiological and pathological conditions. Several miRNAs regulate the protein synthetic pathways and control either ribosome function or the production of ribosomal proteins, thereby having the potential to regulate protein synthesis. For example, miR‐126 targets the IGF‐1 signalling pathway in aging muscle, and inhibition of miR‐126 increases phosphorylation of ribosomal protein S6 in response to IGF‐1.^23^ Similarly, miR‐542‐3p promotes ribosomal stress by targeting small ribosomal proteins in the cytoplasm.^24^ It therefore seems likely that miR‐542‐3p will inhibit protein synthesis although this remains to be established. Another miRNA that targets ribosomal proteins is miR‐10a, but unlike most known functions of miRNAs, miR‐10a increases synthesis of these target proteins by binding to the 5′UTR and increasing translational initiation.^25^

In a screen of miRNAs that were altered in the quadriceps of patients with chronic obstructive pulmonary disease (COPD) compared with controls, we found that miRNAs from the miR‐542/424 cluster located on the X chromosome were elevated.^26^ MiR‐542‐3p targets both the cytoplasmic and mitochondrial ribosomal proteins indicating that it modulates protein synthesis^24^, ^26^; as miR‐424‐5p is expressed from the same locus, we expected that it would target similar or complementary factors. Consistent with this suggestion, *in silico* analysis predicted that miR‐424‐5p would target a range of proteins involved in rRNA transcription (including PolR1A, the largest subunit and catalytic core of RNA polymerase I, and UBTF) and protein synthesis (including IGF‐1, IGF‐1R, RPS6K, and translation initiation factors). We therefore hypothesized that miR‐424‐5p would suppress protein synthesis and promote muscle wasting. To identify appropriate mRNA targets that would regulate these processes in muscle, we used Argonaute2 (Ago2) pull‐down assays. We then determined the effect of miR‐424‐5p on rRNA and protein synthesis *in vitro* and on muscle fibre size *in vivo*. Finally, we determined the association of miR‐424‐5p with muscle mass and function in a separate large cohort of COPD patients, in patients with established ICU‐acquired weakness (ICUAW), those about to go on to the ICU following aortic surgery, and in older people with and without sarcopenia.

## Materials and methods

### Cell culture and transfection

Human skeletal myoblasts from the line LHCN‐M2 were maintained as described by Zhu *et al*.,^27^ and C2C12 myoblasts were cultured as previously described.^28^ For transfection, cells were seeded at a density of 5 × 10^4^/mL seeding 6250 cells in a 96‐well plate and scaling for the appropriate growth area. After 24 h, the cells were transfected with miR‐424‐5p (or the rodent orthologue miR‐322‐5p, hereafter referred to as miR‐424‐5p) or control *miR*Vana™ (ThermoFisher Scientific, Paisley, UK) mimics using lipofectamine 2000 according to the manufacturer's instructions.

### Argonaute2 pull down

C2C12 cells cultured in 10 cm culture dishes were transfected with either miR‐424‐5p mimic or control mimic and cultured for a further 2 days. After washing twice with ice‐cold phosphate buffered saline (PBS), the cells were lysed with cell lysis buffer (CLB, Cell Signalling Technology, Danvers, MA, USA) supplemented with protease inhibitor cocktail (Sigma‐Aldrich, Gillingham, UK). The lysate was pre‐cleared with G‐sepharose beads for 2 h at 4°C then divided into two. Immunoprecipitation was performed by incubating the lysate in 1× CLB, G‐sepharose beads [prepared by pre‐incubation in CLB supplemented with salmon sperm DNA (0.2 mg/mL) and Bovine Serum Albumin (BSA) (1 mg/mL)] with either anti‐Ago2 antibody (Millipore, Billerica, MA, USA) or anti‐IgG antibody, overnight at 4°C under rotation. The beads were washed in CLB, once, IP buffer [50 mM Tris (pH 7.4), 5 mM MgCl_2_, 300 mM NaCl, 0.05% NP40] four times, and PBS once. RNA was extracted from the beads using TRIzol and cDNA synthesis. Data for each cDNA were normalized to glyceraldehyde 3‐phosphate dehydrogenase (GAPDH) in the same sample and analysed as fold enrichment (anti‐Ago2/control IgG) for miR‐424‐5p compared with control.

### Western blotting

Western blotting was performed as previously described.^29^ The primary antibodies used were anti‐UBTF (*Santa Cruz* Biotechnology, Santa Cruz, CA, USA, 1:500 dilution in PBS supplemented with 5% milk, PBS+5%) and anti‐puromycin (Millipore, 1:1000 dilution in PBS+5% milk), and the detection antibody was horse radish peroxidase (HRP)‐conjugated sheep anti‐mouse (GE Healthcare, Amersham, UK, 1:10 000 dilution in PBS+5% milk). The blots were normalized to total protein on the blot by staining with Ponceau S. Blots were digitally scanned, and the image files were analysed using Fiji software. Normalization data are shown in the Supporting Information.

### Assessment of RNA expression

RNA was extracted from muscle samples using the TRIzol method as previously described.^29^, ^30^ RNA from cells cultured in 96‐well plates was extracted using CellAmp Direct RNA Prep Kit (Takara Bio Europe SAS, Saint‐Germain‐en‐Laye, France) according to the manufacturer's instructions. miRNAs were quantified using probes purchased from Applied Biosystems Life Technologies (Paisley UK) as previously described.^31^ miRNA expression was normalized to U6 and RNU48 expression as previously described.^26^ mRNAs and rRNAs were quantified using SYBR green as previously described^32^ and normalized to the geometric mean of β2 microglobulin and GAPDH (mouse and culture samples) or β2 microglobulin and hypoxanthine‐guanine phosphoribosyltransferase (HPRT) for the same sample using primers in the Supporting Information, *Table* S1. Normalization data are shown in the Supporting Information. RNA data are presented as log normalized value (log 2^−ΔCT^) or as linear fold change compared with control (2^−ΔCT^ test/2^−ΔCT^control). Linear fold change was calculated on normalized but unlogged data.

### Protein synthesis assay

LHCN‐M2 cells were seeded and transfected as previously described and 48 h later were serum and leucine starved for 2 h. Cells were placed in leucine‐containing Dulbecco's Modification of Eagle's Medium (DMEM) supplemented with 130 nM of IGF‐1 (Cambridge Bioscience, Cambridge, UK) for 45 min, before the addition of 100 ng/mL puromycin (Sigma‐Aldrich) for 30 min before harvesting. 300ng of protein was added to each well of a 96‐well plate in 200 μL of 50 mM sodium bicarbonate and incubated at 37°C for 2 h. After washing (1× PBS), 200 μL of blocking buffer PBS+(5% BSA) was added and the samples incubated for 30 min at room temperature (RT). The solution was replaced with 100 μL of 100 ng/mL anti‐puromycin (Millipore) diluted in PBS+5% BSA and incubated for 1 h at room temperature. After 2× PBS washes, 100 μL of sheep anti‐mouse (GE Healthcare) was added at a 1/10 000 dilution in PBS+5% BSA as per manufacturer's instructions. The samples were washed 4× in PBS before 100 μL of TMB substrate (Sigma‐Aldrich) was added and the samples incubated for 15 min. The reaction was stopped with 100 μL of stopping solution (Sigma‐Aldrich) and absorbance determined at 450 nm. From each sample, 5 μg of protein was subsequently analysed by western blotting (Supporting Information).

### *In vivo* experiments

#### Cloning of expression vector for miR‐424

A 500 bp fragment of the miR‐322 locus was amplified from mouse genomic DNA by polymerase chain reaction (PCR) using the primers ATAAGATCTGGCTCCACCTGCAGCTCCTGGAAATC and ATAAGATCTGCGCCCCAGCCTAGCCAGGAATAC. The PCR product was cloned into pGEM‐T‐easy, sequenced then digested out of the vector with BglII, and shuttled into the BamHI site of pCAGGS‐EGFP^32^ and the orientation confirmed. This vector, designated pCAGGS‐EGFP‐424, would allow the over‐expression of miR‐424 and EGFP from the same primary transcript, so allow the identification of successfully transfected fibres expressing the miRNA. It also allowed the use of pCAGGS‐EGFP as an empty vector control to identify transfected fibres in the control limbs.

#### Electroporation

Mouse experiments were approved by the Imperial College Ethical Review Process and were licensed by the UK Secretary of State for the Home Office under Project License PPL 70/8297. Five male C57/Bl6 mice (7.5 weeks old) were anaesthetized with 1:1 Hypnorm (VetaPharma, Leeds, UK) and Hypnovel (Roche Products Ltd, Welwyn Garden City, UK); both lower legs were shaved, and 10 U (25 μL) of bovine hyaluronidase (Sigma‐Aldrich) was injected into each *tibialis anterior* (TA).^33^, ^34^ Mice were allowed to partially recover at 37°C and after 2 h were re‐anaesthetized using 5% isofluorane then maintained at 2% isofluorane. The TA muscles were injected with 25 μL of the appropriate plasmid (pCAGGS‐EGFP‐424 into one TA and pCAGGS‐EGFP into the contralateral TA) at 1 μg/μL before electroconductive cream was applied to electrodes that were placed on either side of the TA, separated by approximately 5 mm. Electroporation was performed using 10 pulses of 85 V each for 20 ms, at a frequency of 1 Hz.

Following electroporation, the mice were allowed to recover then were monitored daily for 3 days prior to sacrifice. The TA muscles were removed and weighed before being embedded and sectioned as previously described.^33^ By expressing EGFP‐miR‐424 in one limb and EGFP alone in the contralateral limb, we were able to compare muscles within individual animals.

### Patient cohorts

The samples used in this study have been described and analysed for other miRNAs in previous studies.

#### Chronic obstructive pulmonary disease cohort

The cohort of patients with COPD comes from a larger study that has been described previously.^35^ Briefly, patients were recruited from clinics at the Royal Brompton Hospital. Exclusion criteria included renal, liver, or heart failure or a moderate/severe exacerbation in the preceding 4 weeks. Healthy age‐matched controls were recruited by advertisement. Written informed consent was obtained from all patients, and the protocol was approved by the appropriate research ethics committee (studies 06/Q0404/35 and 06/Q0410/54). PCR for miR‐424‐5p was performed on samples from 57 patients and 17 controls, and the data described in this paper are taken from all patients for whom there was appropriate amplification and suitable replicates (*n* = 49 patients and 16 controls). Demographic data for the cohort are given in the Supporting Information, *Table* S2. Fat‐free mass index (FFMI) was determined by bioelectrical impedance (Bodystat 1500, Bodystat, UK) in patients who had been resting supine for 10 min. Quadriceps strength was measured as supine maximal voluntary contraction as described previously and physical performance as 6 min walk distance according to the American Thoracic Society 2002 guidelines.^36^ A biopsy of *vastus lateralis* in the leg tested for strength was performed under local anaesthetic by the Bergstrom technique on a separate visit to exercise testing and after 20 min rest, but patients had not fasted prior to biopsy.^37^

#### Hertfordshire Sarcopenia Study cohort

The study protocol was approved by the Hertfordshire Research Ethics Committee (study 07/Q0204/68), and all participants gave written informed consent. Muscle mass and strength together with gait speed and a timed‐up‐and‐go (TUG) test were ascertained; Hertfordshire Sarcopenia Study methods have been previously described.^38^ Muscle biopsy was performed by percutaneous conchotome biopsy of the *vastus lateralis* under local anaesthesia after an overnight fast and prior to physiological testing.^39^ Demographic data for these patients are shown in the Supporting Information, *Table* S3.

#### Intensive care unit‐acquired weakness cohort

This cohort has been described previously.^40^ Written informed consent was obtained from study subjects or their next of kin, and the study was approved by the National Research Ethics Committee 10/H0722/9. The muscle biopsies were taken from the rectus femoris by the Bergstrom technique^37^ or as open biopsies. Demographic data are shown in the Supporting Information, *Table* S4.

#### Aortic surgery cohort

Patients undergoing elective aortic surgery at the Royal Brompton Hospital were recruited to the study and provided written informed consent. The study was approved by the National Research Ethics Committee (07/Q0204/68). The principal inclusion criterion was an elective aortic operation requiring admission to the ICU as identified by the surgical team. Exclusion criteria included pre‐existing muscular or neuromuscular disease and malignancy or contraindication to muscle biopsy. Rectus femoris cross‐sectional area (RF_CSA_) was determined by ultrasound^41^ before and 7 days after surgery and used as a measure of muscle loss. An open biopsy of the rectus femoris (to enable direct comparison with the ultrasound data) was taken under general anaesthetic by the surgical team prior to surgery with a second biopsy taken 24 h after surgery using the Bergstrom technique under local anaesthesia. The patients will have been fasted for surgery and rested at the times of both biopsies. Physiological assessment was carried out at a separate visit prior to surgery and biopsy. Demographic data and data associated with the procedure are given in the Supporting Information, *Table* S5.

### Statistical analyses

All RNA data were log transformed to standardize the variance. Statistical analysis was performed in Aabel (Gigawiz). Correlation analysis was performed using Pearson correlation after visual inspection for linear relationships. Student's *t*‐test (normally distributed data) and Mann–Whitney *U* test (non‐parametric data) were used to calculate between group differences for two groups. Differences between multiple groups in the same analysis were calculated by Kruskall–Wallis analysis with post hoc analysis by Mann–Whitney *U* test (non‐parametric data).

## Results

### miR‐424‐5p targets proteins associated with rRNA and protein synthesis

**Figure 1:**
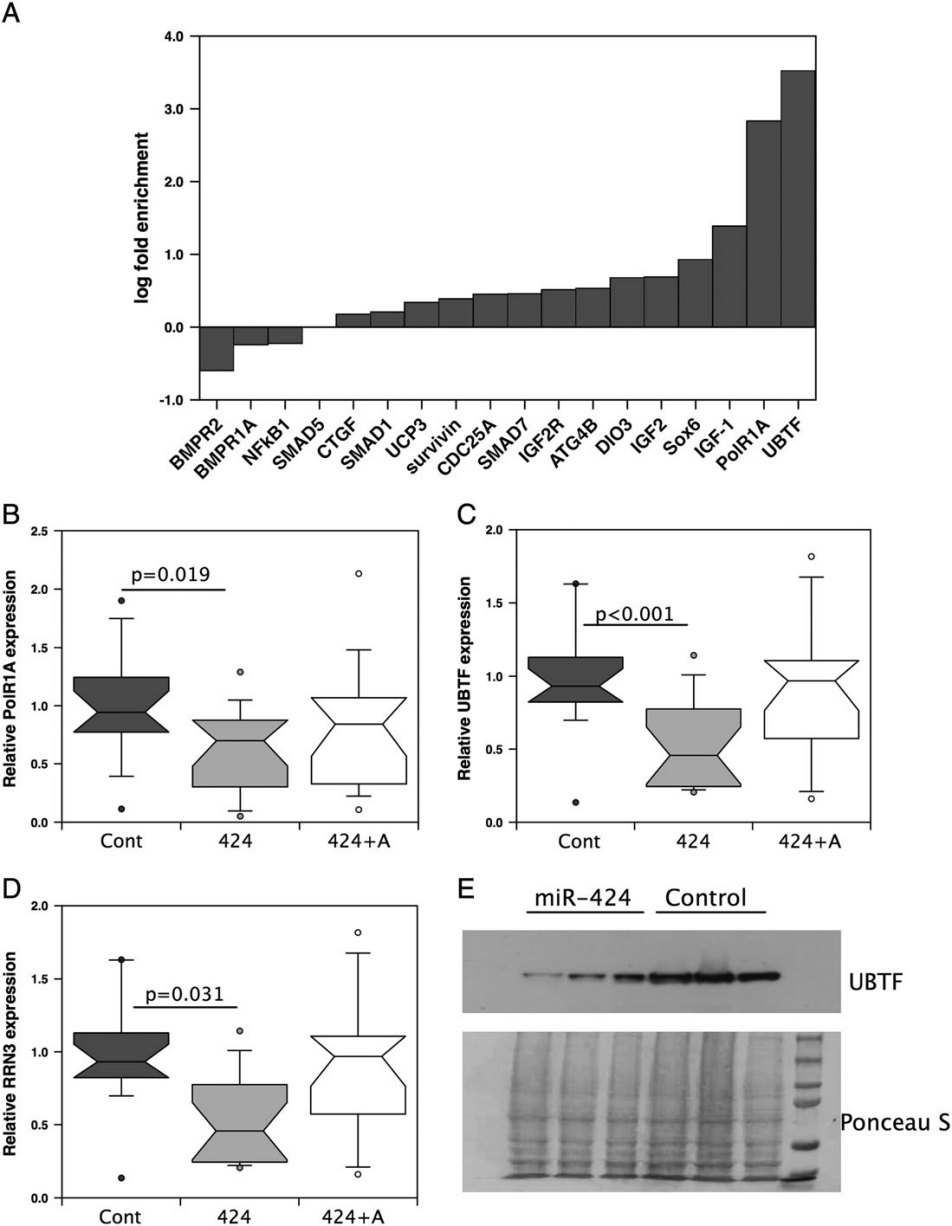
miR‐424‐5p targets components of the RNA Pol1 pre‐initiation complex. (*A*) RNA was immunoprecipitated from C2C12 cells transfected with the rodent miR‐424‐5p orthologue mimic or a control mimic, and the presence of individual mRNAs was quantified by quantitative polymerase chain reaction as described in the Materials and methods section. The amount of each RNA was normalized to glyceraldehyde 3‐phosphate dehydrogenase and fold enrichment determined by comparing normalized mRNA between miR‐424‐5p and control transfected samples immunoprecipitated with anti‐Argonaute2. Data are presented as log ‘fold enrichment’ because of the scale of enrichment in PolR1A and UBTF. (*B*–*E*) LHCN‐M2 cells were transfected with control mimic (Cont), miR‐424‐5p mimic (424), or miR‐424‐5p mimic and antagomiR (424 + A). Two days later, RNA or protein was extracted and the expression of PolR1A (*B*), UBTF (*C*), and RRN3 (*D*) mRNAs or UBTF protein (*E*) was quantified. The expression of RNAs was normalized to the geometric mean of β2 microglobulin and hypoxanthine‐guanine phosphoribosyltransferase (Supporting Information), and UBTF protein was normalized to Ponceau S staining (E and in the Supporting Information). Data are presented as linear relative change. Transfection suppressed the expression of all three microRNAs and reduced the amount of UBTF protein in cells. Expression data are from three independent experiments of six individual transfections assayed in duplicate. Box and whisker plots show median and interquartile range with the whiskers to the 90th centiles and outliers shown. IGF, insulin‐like growth factor; mRNA, messenger RNA; UBTF, upstream binding transcription factor

Bio‐informatic analysis was carried out using miRwalk 2.0^42^ selecting all databases and searching for interactions with all mRNA 3′‐UTRs. RNAs were selected as potential targets if they were predicted to interact with the miRNA by at least five databases. This approach showed that, in addition to the previously confirmed targets,^43^, ^44^, ^45^ miR‐424‐5p was predicted to target a number of proteins involved in protein synthesis or in the inhibition of catabolic processes (Supporting Information, *Table* S6). To determine whether there was a direct interaction between the miRNA and some of the predicted mRNAs (i.e. targeting), Ago2 pull‐down assays were performed using RNA from mouse C2C12 cells transfected with miR‐322‐5p (the rodent orthologue of miR‐424‐5p, hereafter referred to as miR‐424‐5p for simplicity). Quantitative PCR for predicted targets showed enrichment (linear fold increase vs. control) for the known targets (SMAD7^46^ and CDC25A^44^) of approximately two‐fold compared with transfection with control miR mimic. In addition to enrichment of these targets, there was an approximate 3000‐fold enrichment of UBTF and a 700‐fold enrichment of PolR1A, suggesting that the miRNA binds to these mRNAs and therefore may target the expression of proteins associated with rRNA synthesis (*Figure* 1A).

The marked enrichment for PolR1A and UBTF suggested that the miRNA would interfere with the formation of the RNA Pol I PIC that is required for the initiation of rRNA transcription. We therefore determined whether miRNA‐424‐5p would reduce the expression of these RNAs in human LHCN‐M2 myoblasts. Transfection of LHCN‐M2 myoblasts with miR‐424‐5p reduced the expression of PolR1A and UBTF, as well as RRN3 (which was predicted to be an miR‐424‐5p target by two databases) another component of the RNA Pol I PIC, and in each case, this was partially or completely reversed by co‐transfection with an antagomir (*Figure* 1B–D). As the largest reduction was in UBTF, we determined the effect of the miRNA on UBTF protein levels and found a marked reduction in protein in miR‐424‐5p transfected cells compared with those transfected with a control miRNA (*Figure* 1E and Supporting Information, *Figure* S1).

### miR‐424‐5p reduces rRNA and protein synthesis in muscle cells

**Figure 2:**
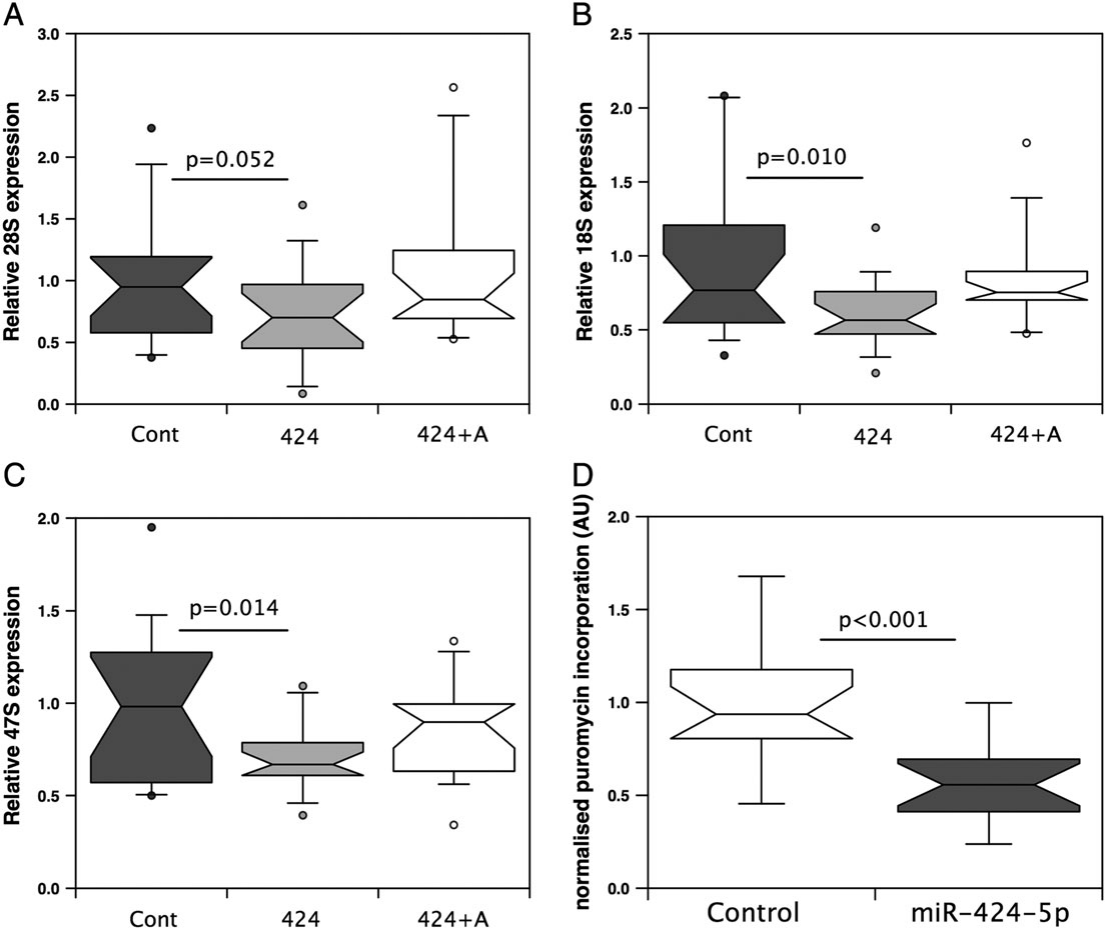
miR‐424‐5p reduces the ribosomal RNA (rRNA) levels and protein synthesis in myoblasts. LHCN‐M2 cells were transfected with control mimic (Cont), miR‐424‐5p mimic (424), or miR‐424‐5p mimic and antagomiR (424 + A). Two days later, RNA was extracted and the expression of 28S rRNA (*A*), 18S rRNA (*B*), and 47S rRNA (*C*) was quantified or the rate of new protein synthesis was determined by puromycin incorporation (*D*). The expression of RNAs was normalized to the geometric mean of β2 microglobulin and hypoxanthine‐guanine phosphoribosyltransferase for the same sample and is presented as linear relative change. miR‐424‐5p reduced the expression of the 18S and 47S rRNAs and suppressed puromycin incorporation into new polypeptides. Expression data are from three independent experiments of six individual transfections assayed in duplicate. Box and whisker plots show median and interquartile range with the whiskers to the 90th centiles and outliers shown.

A reduction in levels of the rRNA PIC would imply a reduction in rRNA production in the cell. We therefore quantified both mature 28S and 18S rRNAs as well as the primary 47S rRNA transcript from which these rRNAs are processed. Both 18S and 47S rRNAs were reduced relative to standard housekeeping genes (GAPDH and β2 microglobulin; see Supporting Information for normalization data), and again, the reduction was reversed by co‐transfection with the antagomiR. Median 28S rRNA expression was lower in miR‐424‐5p transfected cells than in cells transfected with the control or in the antagomiR co‐transfected cells, but this difference did not meet statistical significance (*Figure* 2A–C).

A reduction in rRNA ought to be associated with a reduction in maximal protein synthesis and reflect a reduction in the capacity of the protein synthetic machinery. We therefore quantified protein synthesis in the presence of leucine, IGF‐1, and serum using puromycin incorporation. Consistent with a reduction in protein synthetic capacity, miR‐424‐5p suppressed puromycin incorporation into nascent polypeptides (*Figure* 2D and Supporting Information, *Figure* S2).

### miR‐424 causes muscle wasting in mice

**Figure 3:**
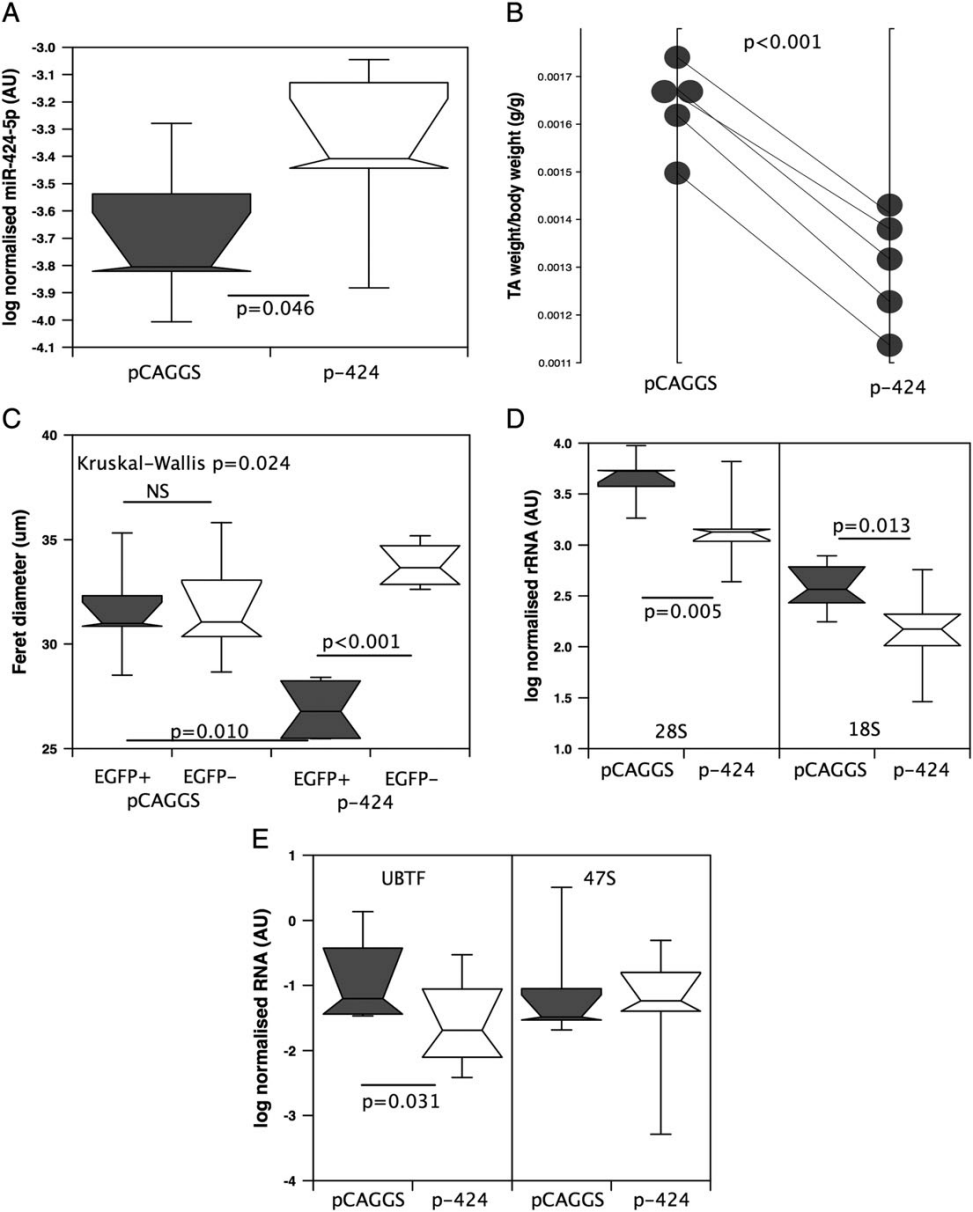
Over‐expression of miR‐424 reduces muscle size and the expression of upstream binding transcription factor (UBTF) and ribosomal RNAs (rRNAs). The *tibialis anteriors* (TAs) of mice (*n* = 5) were electroporated with pCAGGS‐EGFP (pCAGGS) or pCAGGS‐EGFP‐424 (p‐424). Three days later, the mice were sacrificed and the muscle dissected out and weighed. The expression of miR‐424‐5p (*A*) was higher in the pCAGGS‐EGFP‐424 transfected leg than in the control leg. (*B*) TA weight normalized to body weight was lower in the miR‐424 expressing leg compared with the contralateral control. (*C*) Fibre diameter was determined for the transfected fibres and non‐transfected fibres in both legs of four of the mice because of ice crystal formation in one sample. EGFP‐positive fibres from the miR‐424 expressing leg were smaller than EGFP‐negative fibres in the same leg and both EGFP positive and EGFP negative fibres in the control transfected leg. Electroporation of pCAGGS‐EGFP‐424 reduced the expression of 28S, 18S (*D*) and of UBTF (*E*) compared with electroporation of pCAGGS‐EGFP. 47S rRNA did not differ between the samples (*E*). mRNA and rRNA data were normalized to the expression of β2 microglobulin and GAPDH for the same sample then logged. miRNA data were normalized to the expression of U6 and RNU48 in the same sample then logged. Box and whisker plots show median and interquartile range with the whiskers to the 90th centiles and outliers shown.

To determine whether over‐expression of miR‐424 would promote muscle wasting *in vivo*, we generated a miR‐424 expression vector by cloning a 500 bp fragment of the mouse orthologue of the miR‐424 locus (miR‐322) into the 3′‐UTR of the EGFP gene in pCAGGS‐EGFP to generate pCAGGS‐EGFP‐424. Electroporation of either vector resulted in EGFP expression in >50% of the fibres and was associated with 1.5% of the fibres showing centralized nuclei, consistent with a low level of fibre damage (Supporting Information, *Figure* S3) with no detectable difference in either parameter between the two vectors. miR‐424 expression in the TA electroporated with pCAGGS‐EGFP‐424 was higher than in the contralateral TA electroporated with pCAGGS‐EGFP (*Figure* 3A), indicating appropriate processing of the miRNA.

Electroporation of pCAGGS‐EGFP‐424 into the TA of mice caused a marked reduction in muscle weight (21%) 3 days later compared with the contralateral TA electroporated with pCAGGS‐EGFP. Furthermore, quantification of fibre feret diameter and fibre area showed that there was a reduction in the area of fibres in the TA that were successfully transfected with pCAGGS‐EGFP‐424 (as determined by EGFP fluorescence) compared with untransfected fibres in the same leg and fibres successfully transfected with pCAGGS‐EGFP in the contralateral leg (*Figure* 3B and 3C and Supporting Information, *Figure* S3).

Expression of 18S and 28S rRNAs was reduced in the muscle electroporated with pCAGGS‐EGFP‐424 compared with the contralateral leg, as was the expression of UBTF (*Figure* 3D–E). However, there was no significant reduction in the expression of PolR1A, and the expression of RRN3 was not detectable in enough samples to generate a meaningful result.

### miR‐424‐5p is increased in patients with COPD and associated with muscle function

**Figure 4:**
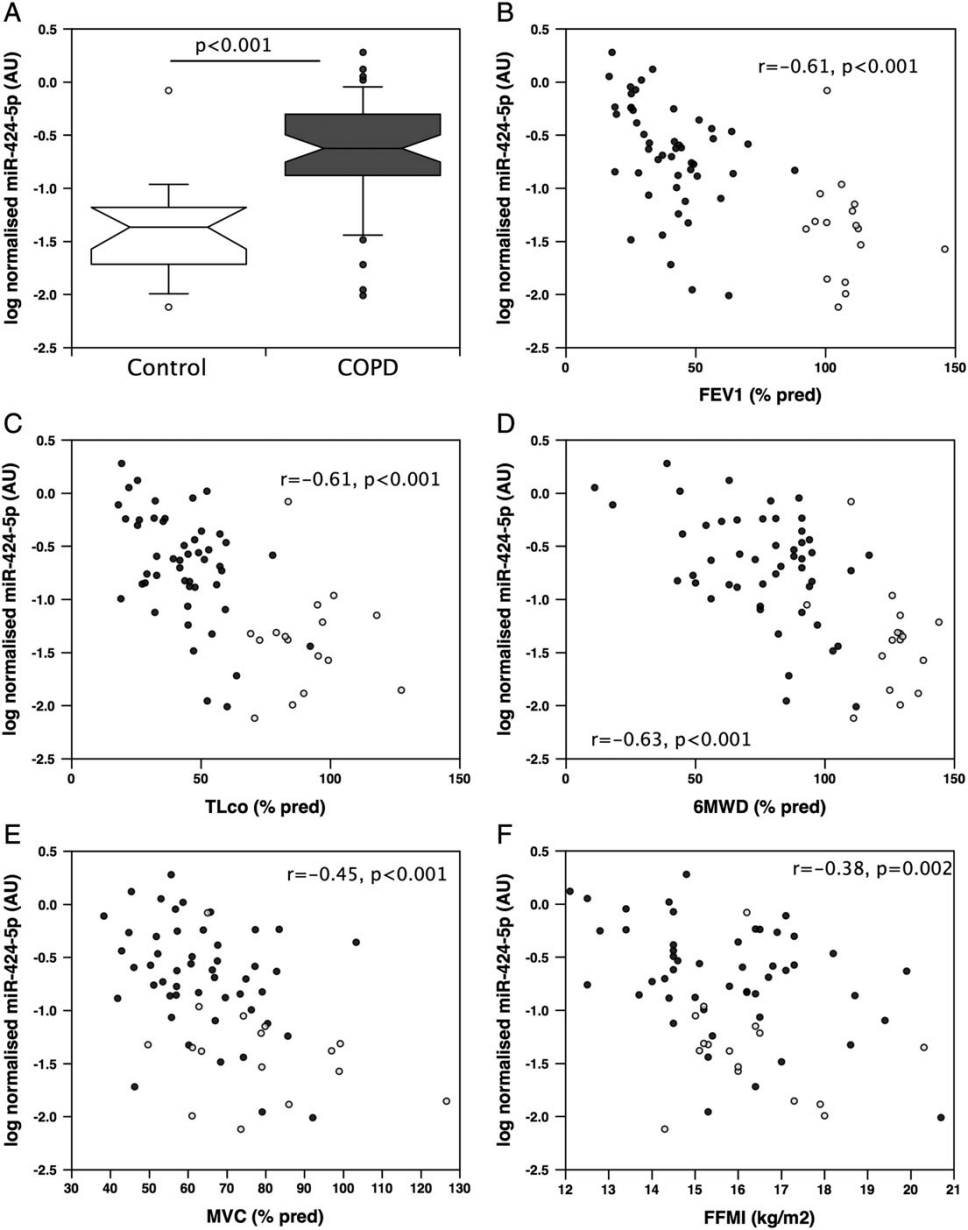
miR‐424‐5p is elevated in patients with chronic obstructive pulmonary disease (COPD) and associated with disease severity and with muscle mass and function. miR‐424‐5p was quantified in quadriceps biopsies from patients with COPD (*n* = 49) and controls (*n* = 16). Quadriceps miR‐424‐5p was elevated in the COPD patients (*A*) (*t*‐test) and was inversely correlated with disease severity measured either as FEV_1_% (*B*) or as TL_CO_% (*C*). Quadriceps miR‐424‐5p was also inversely proportional to muscle function measured both as 6 min walk distance % predicted (6MWD%, *D*) and as maximal voluntary contraction % predicted (MVC%, *E*) as well as with fat‐free mass index (FFMI, *F*). miRNA data were normalized to the expression of U6 and RNU48 for the same sample then logged. Pearson correlations were performed, and the values shown are for the whole cohort. The box and whisker plot shows median and interquartile range with the whiskers to the 90th centiles and outliers shown.

To confirm the elevation of miR‐424‐5p in the muscle of COPD patients, the expression of this miRNA was determined in quadriceps from a larger cohort of patients of both sexes and all Global Initiative for Chronic Obstructive Lung Disease stages (*n* = 49) and in age‐matched controls (*n* = 16). The demographics of this cohort are given in the Supporting Information, *Table* S2. Consistent with a diagnosis of COPD, patients had reduced lung function (FEV_1_% predicted and TL_CO_% predicted) and greater smoking history compared with controls (Supporting Information, *Table* S2). Patients had reduced physical performance (6MWD%) and strength compared with controls (Supporting Information, *Table* S2), but neither BMI nor FFMI was different from controls. This analysis showed that miR‐424‐5p was approximately four‐fold (linear) higher in patients than in age‐matched controls (*P* < 0.001, *Figure* 4A). miR‐424‐5p expression was tightly associated with miR‐542‐3p and miR‐542‐5p, suggesting that expression is driven by the same promoter or by one under similar control (Supporting Information, *Figure* S4). Similar to these miRNAs, quadriceps miR‐424‐5p expression was associated with disease severity (*Figure* 4B and 4C) measured as FEV_1_% predicted (*r* = −0.61, *P* < 0.001 in the whole cohort and *r* = −0.41, *P* = 0.003 in the patients alone) and as TL_CO_ % predicted (*r* = −0.61, *P* < 0.001 in the whole cohort and *r* = −0.48, *P* < 0.001 in the patients alone). Quadriceps expression of miR‐424‐5p was also inversely proportional to physical performance (*Figure* 4D and 4E) measured as 6 min walk distance (*r* = −0.63, *P* < 0.001 in the whole cohort and *r* = −0.46, *P* < 0.001 in the patients alone) and to strength measured as maximal voluntary contraction (*r* = −0.45, *P* < 0.001 in the whole cohort and *r* = −0.33, *P* = 0.021 in the patients alone). miR‐424‐5p was more strongly associated with FFMI than miR‐542‐3p/5p, as the association was present not only in the whole cohort (*Figure* 4F, *r* = − 0.38, *P* = 0.002) but also in patients considered as a separate group (*r* = −0.41, *P* = 0.004).

### miR‐424‐5p is increased in sarcopenia

**Figure 5:**
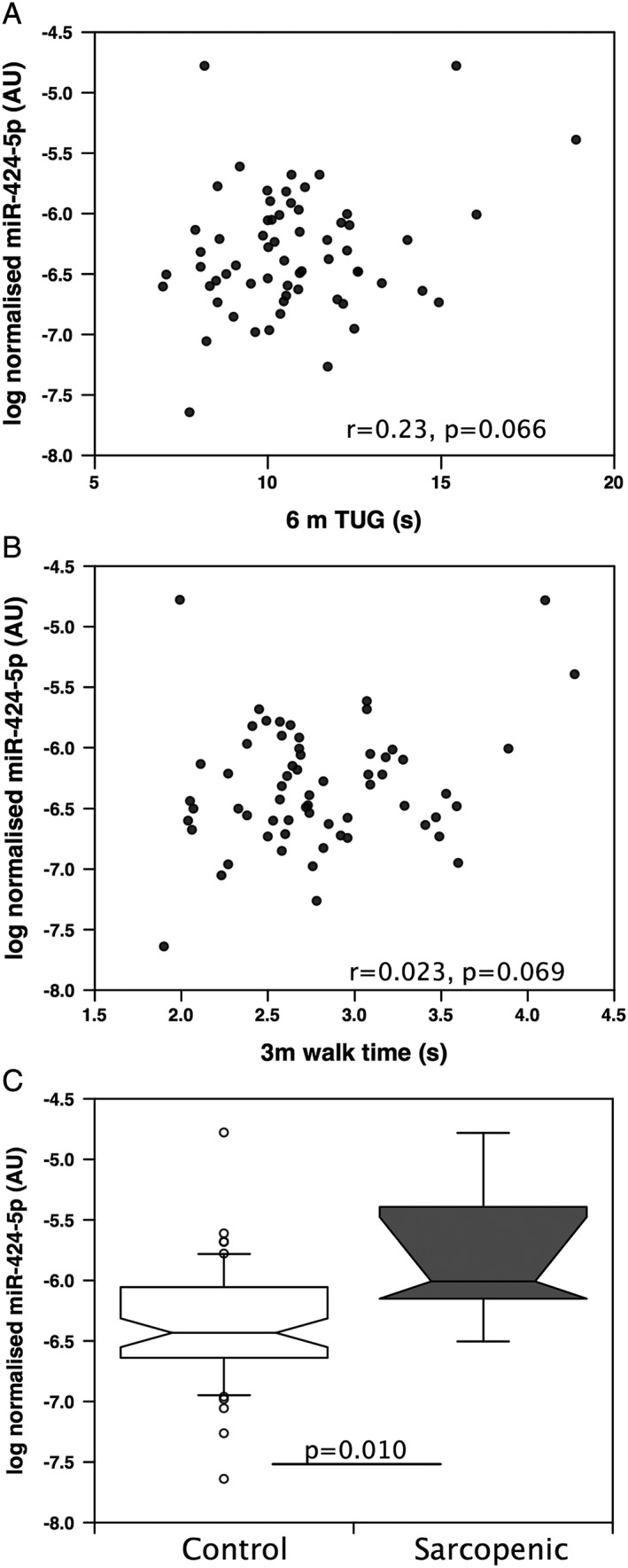
miR‐424‐5p is elevated in sarcopenia. miR‐424‐5p was quantified in the quadriceps muscle of individuals from the Hertfordshire Sarcopenia Study (*n* = 65). Any association of the microRNA with 6 m timed up and go (6 m TUG, *A*) and 3 m walk time (*B*) did not reach statistical significance, but miR‐424‐5p was elevated in sarcopenic individuals (*n* = 5) compared with non‐sarcopenic individuals (*n* = 60, *C*). miRNA data were normalized to the expression of U6 and RNU48 for the same sample then logged. The box and whisker plot shows median and interquartile range with the whiskers to the 90th centiles and outliers shown.

To determine whether similar associations occurred in older individuals from the general population, we determined the expression of miR‐424 in samples from the Hertfordshire Sarcopenia Study (*n* = 64). Five of these individuals were defined as sarcopenic as determined by the European Working Group on Sarcopenia in Older People criteria. Consistent with these criteria, FFMI and physical performance [timed up and go (6 m TUG) and 3 m gait speed] in sarcopenic individuals were lower than in the non‐sarcopenic cohort (Supporting Information, *Table* S3). In these individuals, associations between miR‐424‐5p expression in the quadriceps and reduced physical performance measured as both 6 m TUG and 3 m gait speed did not reach statistical significance (*r* = 0.23, *P* = 0.066 and *r* = 0.23, *P* = 0.069, respectively, *Figure* 5), nor was there any association of the miRNA with FFMI, but miR‐424‐5p expression was higher in those defined as sarcopenic by the European Working Group on Sarcopenia in Older People criteria (*P* = 0.01) than in non‐sarcopenic individuals (*Figure* 5). However, this observation is limited by the fact that only five individuals were classified as sarcopenic within the whole cohort.

### miR‐424‐5p is associated with muscle loss in patients in the ICU

**Figure 6:**
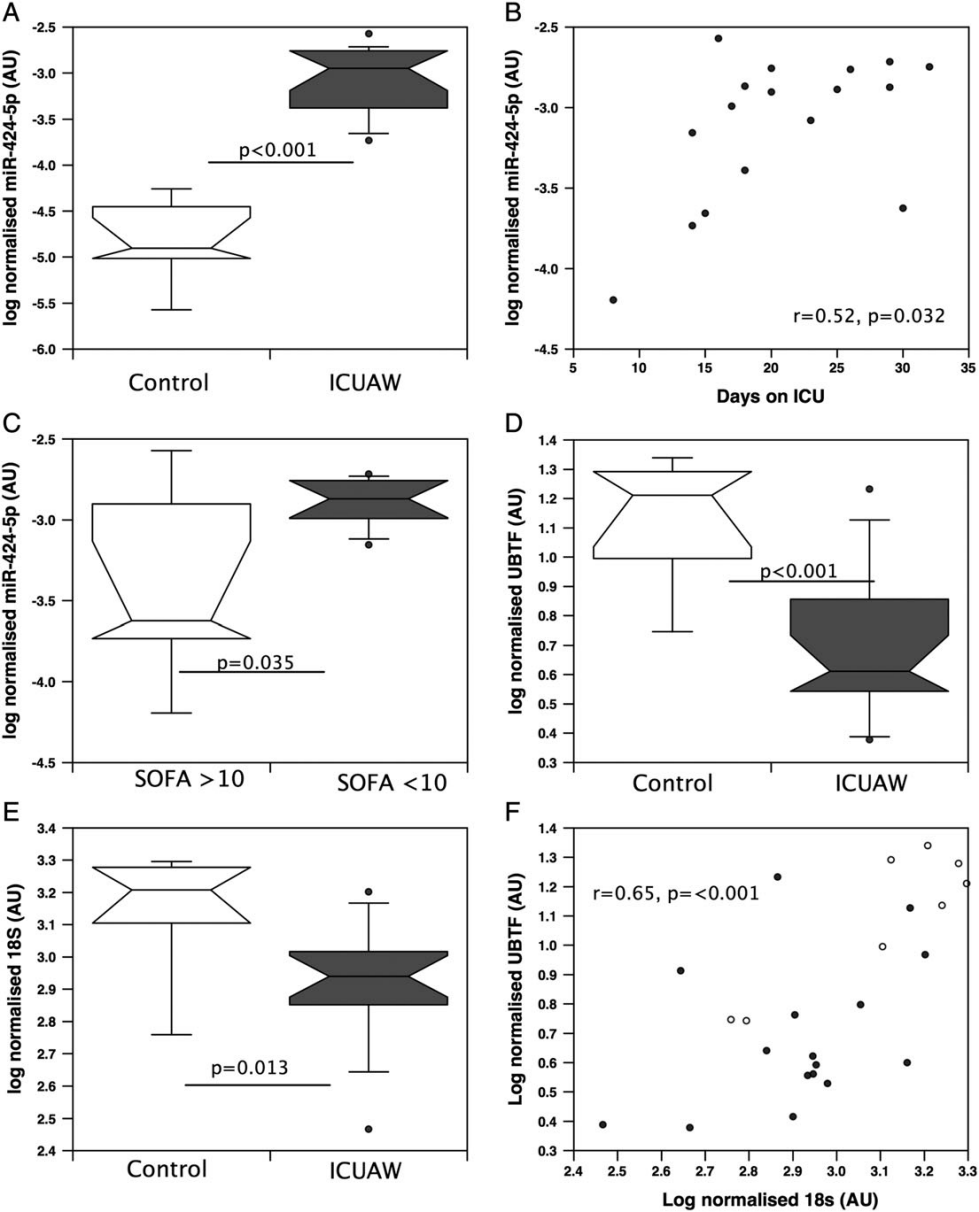
Quadriceps miR‐424‐5p is elevated, and upstream binding transcription factor (UBTF) and 18S ribosomal RNA (rRNA) are reduced in patients with intensive care unit (ICU)‐acquired weakness (ICUAW). miR‐424‐5p was quantified in muscle biopsies from patients with ICUAW (*n* = 17) and controls (*n* = 7). miR‐424‐5p was elevated in patients with ICUAW (*A*) and associated with length of time on the ICU (*B*) and higher in those with a Sepsis‐related Organ Failure Assessment (SOFA) score <10 compared with those with a SOFA score >10 (*C*). UBTF was suppressed in patients with ICUAW compared with controls (*D*) as was the expression of the 18S rRNA (*E*). 18S rRNA correlated with UBTF in the cohort as a whole (*F*). mRNA and rRNA data were normalized to the expression of β2 microglobulin and hypoxanthine‐guanine phosphoribosyltransferase for the same sample then logged. miRNA data were normalized to the expression of U6 and RUN48 for the same sample then logged. All comparisons were made by Mann–Whitney *U* test and Pearson correlation. The box and whisker plots show median and interquartile range with the whiskers to the 90th centiles and outliers shown.

Expression of miR‐424‐5p was markedly increased (approximately 50‐fold, *P* < 0.001, *Figure* 6A) in the quadriceps of patients with established ICUAW (*n* = 17) compared with controls (*n* = 7). Muscle layer thickness (in those with a measurement *n* = 12 patients and 5 controls) was reduced in patients compared with controls (Supporting Information, *Table* S4). Muscle expression of miR‐424‐5p was associated with days spent in the ICU (*r* = 0.52, *P* = 0.032) and although the association between Sepsis‐related Organ Failure Assessment (SOFA) score on the day of biopsy and miR‐424‐5p did not reach statistical significance, in patients with a SOFA score >10, miR‐424‐5p was higher compared with those with a SOFA score <10 (*Figure* 6B and 6C). As the ICUAW group showed the largest increase, we determined the expression of UBTF (*Figure* 6D), PolR1A, and RRN3 in the same muscle samples. There was a significant reduction in the expression of UBTF in these patients compared with controls, but although median PolR1A and RRN3 expression were lower in the patients than in the controls, this difference did not reach statistical significance. In these patients, 18S rRNA but not 28S rRNA (*Figure* 6E) was reduced compared with controls leading to a reduction in the 18S:28S rRNA ratio. Furthermore, the expression of UBTF, PolR1A, and RRN3 was positively associated with the expression of the 18S rRNA but not with the 28S rRNA (*r* = 0.65, *P* < 0.001 (*Figure* 6F); *r* = 0.47, *P* = 0.022; and *r* = 0.51, *P* = 0.011 with 18S, respectively).

**Figure 7:**
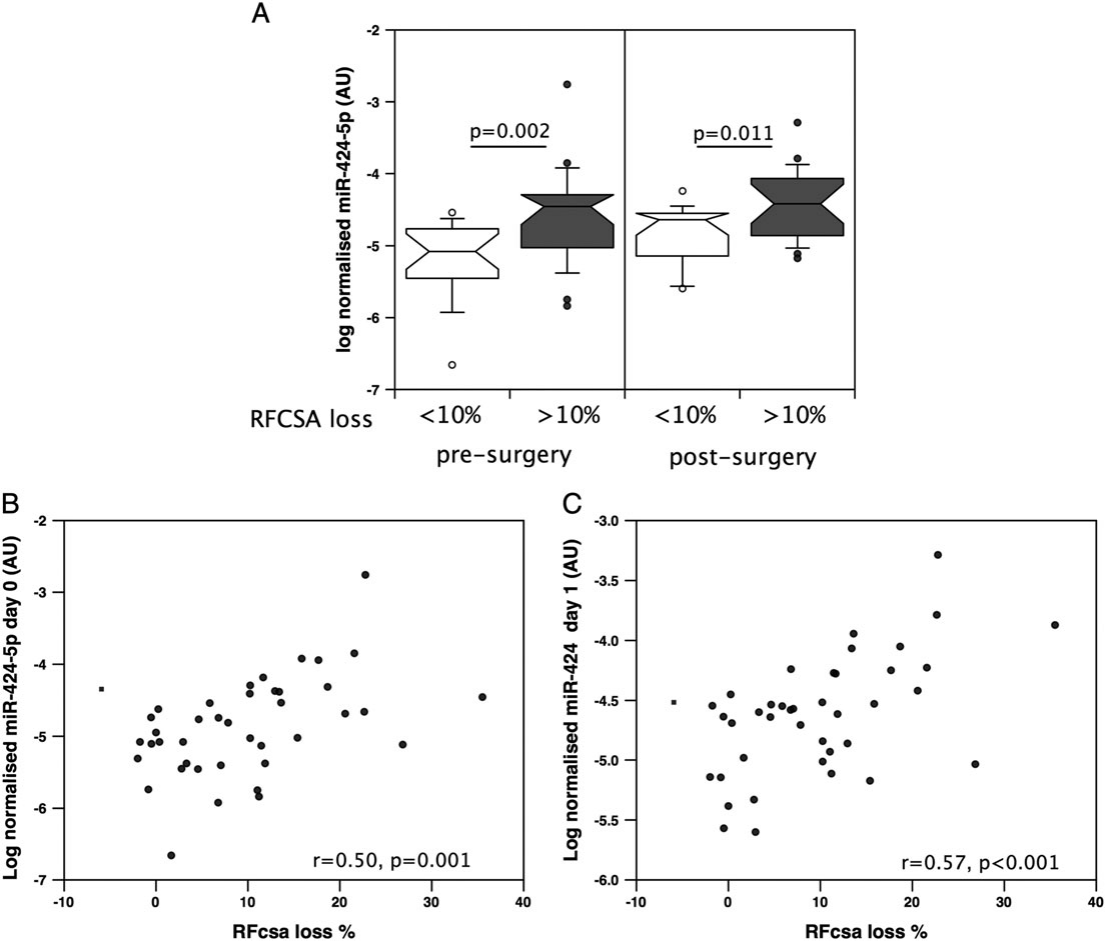
miR‐424‐5p is associated with muscle loss in patients undergoing aortic surgery. miR‐424‐5p was quantified in the rectus femoris of patients (*n* = 40) undergoing aortic surgery on the day of surgery (Day 0) and 24 h after surgery (Day 1). In patients who lost >10% of the cross‐sectional area of their rectus femoris (RF_CSA_), miR‐424‐5p was higher than in those who lost <10% (*A*) on both days and was correlated with the amount of muscle that the patients would lose over the subsequent 7 days (*B*, Day 0, and *C*, Day 1). miRNA data were normalized to the expression of U6 and RNU48 for the same sample then logged. The box and whisker plots show median and interquartile range with the whiskers to the 90th centiles and outliers shown.

Although our mouse data show that miR‐424‐5p can promote muscle loss in response to over‐expression following electroporation, the human studies only show that miR‐424‐5p is elevated in patients with a condition where muscle mass is reduced but do not demonstrate whether this miRNA is associated with the loss of muscle. Consequently, to determine whether higher expression of miR‐424‐5p may promote muscle loss in humans, we determined the expression of miR‐424‐5p in the quadriceps of patients about to undergo aortic surgery. We have previously shown that half of these patients will lose 10% of their RF_CSA_ over the subsequent 7 days. Patients who lost >10% RF_CSA_ had similar rectus femoris muscle size and strength before surgery, but 7 days after surgery, their RF_CSA_ was significantly smaller than in those who did not lose >10% RF_CSA_. These patients also showed a greater reduction in their relative strength 7 days after surgery than those who maintained RF_CSA_ (Supporting Information, *Table* S5). In the patients who lost >10% RF_CSA_, miR‐424‐5p expression was higher before surgery than in those who did not (*P* = 0.002, *Figure* 7A). Indeed, there was a direct correlation of miR‐424‐5p expression with the amount of muscle that would be lost in the following 7 days (*r* = 0.50, *P* = 0.001, *Figure* 7B). In biopsies taken 24 h after surgery, miR‐424 increased compared with prior to surgery (*P* = 0.007) in the cohort as a whole, but this increase was only significant in those who lost <10% RF_CSA_ (*P* = 0.012, *Figure* 7A) when the groups were considered independently. However, the expression of miR‐424‐5p remained higher in the wasters than in the non‐wasters (*P* = 0.011) at this later time point, and the expression was still associated with muscle loss over the 7 days (*r* = 0.57, *P* < 0.001, *Figure* 7C).

## Discussion

The study described here provides both mechanistic and association studies consistent with a role for this miRNA in the loss of muscle mass. Our *in vitro* and animal data show that miR‐424‐5p targets a fundamental process in cell biology, the synthesis of rRNA, by reducing the expression of UBTF, PolR1A, and RRN3 in muscle cells. Furthermore, we show that transfection of this miRNA is sufficient to cause a reduction in maximal protein synthesis. *In vivo,* over‐expression of miR‐424 can cause a marked reduction in fibre diameter. Together, these data show that the miRNA is capable of generating an environment in the cell that can lead to a reduction in muscle size and that reducing the capacity of the protein synthetic machinery is likely to be a component of this process. Our data from human studies show that the miRNA is elevated in conditions where muscle wasting occurs (COPD, sarcopenia, and ICUAW) and that the expression of this miRNA is associated with muscle wasting in patients admitted to the ICU following aortic surgery.

Our findings are consistent with previous reports of increased and reduced ribosome synthesis during muscle hypertrophy and atrophy, respectively. In studies of mechanical overloading in mice, an increase in rRNA content and the mechanisms driving rRNA synthesis have been observed.^47^, ^48^, ^49^, ^50^ For example, increased Pol I expression and association with ribosomal DNA has been reported in response to functional overload in mice at the onset of hypertrophy.^49^ Consistent with increased Pol I activity, the expression of rRNAs was increased, indicating an increase in ribosome production. The increase in Pol I activity in response to resistance exercise or mechanical loading is regulated at least in part by mTOR promoting the association of Pol I in the promoter regions of ribosomal DNA^51^ and by phosphorylating UBTF.^52^ In humans, increased ribosome synthesis has been noted following resistance exercise. For example, an 8 week resistance training protocol increased both precursor and mature rRNA in normal healthy individuals, and the increase in muscle CSA was directly proportional to the amount of total RNA measured as ng/mg tissue.^53^ Furthermore, the increase in rRNA was accompanied by increased UBTF phosphorylation indicative of mTOR activity and increased UBTF activity. However, conversely, in atrophy following denervation, one study has shown a reduction in ribosome synthesis despite increased mTOR signalling. This latter paper showed a reduction in the expression of TAF1a one of the transcription factors required for rRNA production but not of UBTF suggesting that this reduction in ribosome synthesis was not a consequence of elevated miR‐424‐5p.^54^ A marked reduction in UBTF expression and ribosome biogenesis is also seen in cultured rat muscle cells as they withdraw from the cell cycle and differentiate.^55^

Alterations in ribosome content would lead to a reduction in synthetic capacity (anabolic resistance), a factor identified as a significant contributor to the loss of muscle mass in both chronic disease and with normal aging.^14^, ^18^, ^23^ Consistent with this notion, we found that miR‐424‐5p suppressed IGF‐1 and leucine stimulated protein synthesis. This reduction could occur as a result of a loss of the capacity of the protein synthetic system, a loss of sensitivity of the protein synthetic machinery to anabolic stimuli, or both. The association of miR‐424‐5p with muscle loss following aortic surgery and with FFMI in patients with COPD together with its ability to reduce rRNA synthesis suggest that a reduction in the capacity of the system as a result of reduced UBTF and thereby rRNA synthesis contributes the loss of muscle mass in these patients.

Such flexibility in ribosome biogenesis is perhaps unsurprising and is found in numerous other systems. Ribosomes are energetically expensive to synthesize as they require the co‐ordinated synthesis of 4 rRNAs and approximately 80 proteins as well as multiple other factors that control ribosome assembly. As a result, ribosome biogenesis is tightly controlled and co‐ordinated with cell proliferation.^7^ Nucleolar stress and/or reduced rRNA synthesis is seen in yeast in response to starvation^56^ and serum starvation in mammalian cells, as well as in response to many other stresses including ultraviolet damage.^57^, ^58^ Conversely in proliferating cells, there is a marked increase in rRNA synthesis as evidenced by an increase in the size of the nucleoli. miR‐424 has been shown to target cell cycle components to suppress cell proliferation, these effects will be complemented by the suppression of rRNA synthesis. Interestingly, hypercapnia has been shown to suppress rRNA synthesis in muscle cells.^59^

As described, anabolic resistance can also result from a reduction in the sensitivity of the muscle to anabolic signalling, and previous studies have identified such a reduction in the sensitivity of insulin and IGF‐1 signalling, inhibition of Akt/mToR by inflammatory signals,^60^, ^61^ and lipotoxicity.^62^ Interestingly, miR‐424 may also contribute to this component of anabolic resistance, as previous studies have shown that it targets the IGF1 receptor, and one of the most enriched targets identified by our study was IGF‐1 itself.

The observation that quadriceps miR‐424 expression is proportional to classical markers of primary disease severity (TL_CO_ in COPD, LVEF in patients undergoing aortic surgery, and days spent in the ICU in patients with critical illness) suggests a physiological role in the disease process. One possibility is that expression of the miRNA contributes to repair. The muscle is used as a source of amino acids for tissue repair and for inflammatory responses where rapidly dividing cells require amino acids and energy.^8^ As a consequence, glutamine is released from the muscle in patients with trauma and those in the ICU^63^, ^64^ but is also likely to be an important role for muscle under more normal physiological situations where the loss of muscle is much less noticeable. For example, during mammalian evolution, the provision of glutamine to the immune system would need to have occurred in response to infection regardless of the nutritional state of the animal. As muscle makes up approximately 40% of body mass, the amino acid reserve would be sufficient under most conditions and could be readily replenished by feeding at the next opportunity. Such a use of muscle would require flexibility in the rate of synthesis of rRNA and ribosomes in response to inflammatory signals or to other markers of systemic stress. One component of this mobilization would need to be suppression of muscle protein synthesis reducing the usage of circulating amino acids by a ‘non‐essential’ tissue, whereas the other component would be an increase in protein breakdown thereby increasing amino acid supply. Consistent with a role in coordinating these processes, previous studies have shown that miR‐424 inhibits the expression of SMAD7 thereby increasing the sensitivity of cells to transforming growth factor‐β (TGF‐β)‐type ligands.^46^ In the muscle, these ligands include myostatin, a protein known to promote muscle catabolism.^65^ It therefore seems possible that the elevation of miR‐424‐5p contributes to both reduced protein synthesis and increased protein degradation.

Consistent with this notion of a co‐ordinated miRNA response to physiological stress, we show that miR‐424 expression is tightly correlated with miR‐542‐3p and miR‐542‐5p expression (miRNAs located within the same 5 kb genomic locus), suggesting that they are co‐ordinately expressed. We have previously shown that mir‐542‐3p and mir‐542‐5p also promote muscle wasting and muscle dysfunction.^26^ These miRNAs modulate the TGF‐β signalling system by targeting SMAD7, as well as a set of phosphatases that inhibit SMAD activity and promote mitochondrial dysfunction by inhibiting mitochondrial ribosome synthesis. miR‐542‐3p has also been shown to target the production of small ribosomal proteins and is therefore likely to inhibit protein synthesis.^24^ Together, the elevation of miR‐542‐3p/5p with miR‐424 is likely to promote muscle wasting at least in part by reducing the number of functioning ribosomes.

### Critique of the method

In this study, we demonstrate that miR‐424 is elevated in a range of conditions for which muscle wasting is a common co‐morbidity. We also show in cell and *in vivo* systems that miR‐424 can inhibit rRNA and protein synthesis and promote muscle wasting. Consequently, we show that the miRNAs can cause muscle wasting in an animal model and are elevated at a time when muscle wasting is occurring in humans. However, whilst this miRNA may be able to promote muscle wasting in response to disease, it does not show a very strong association with muscle mass. Muscle wasting in chronic conditions is a complex phenomenon as it reflects a change in balance between multiple processes involved in the synthesis and breakdown muscle. Whilst the relative rates of these processes must be balanced within an individual, the absolute rates of each process will vary between individuals and will be governed by the genetics and epigenetics of the individual. We therefore hypothesize that miR‐424‐5p contributes to the loss of muscle by providing a driving force, reducing the rate of protein synthesis from individual nuclei and increasing the sensitivity of cells to TGF‐β family ligands. However, the rate of loss of nuclei and their rate of replacement by satellite cell recruitment will be important and is likely to be varied between individuals. For example, we have previously shown that miRNAs from imprinted loci that affect myoblast proliferation and stem cell pluripotency are associated with muscle mass and strength in patients with COPD but not in normal individuals.^66^, ^67^ These data suggest that it is only in response to a systemic stress that differences in regeneration rates lead to altered susceptibility to atrophy. We also hypothesize that miR‐424 contributes to the response to that systemic stress.

The measurements of muscle mass used in this study do not take into account changes in the quality of muscle or the fat infiltration and fibrosis that also occur. It is therefore possible that the amount of muscle tissue loss that occurs is larger than we have accounted for. However, the most important association that we observe in patients is that between miR‐424‐5p and muscle loss in the aortic surgery cohort. The rate of the loss in this study is probably too fast for either of these processes to be significant contributors.

One major problem with analysing factors that alter rRNA synthesis is that all RNA quantifications are performed relative to a housekeeping component. These are chosen on the basis that they do not vary between samples and allow for variations in RNA extraction and reverse transcription to be taken into account. This stability is established by assuming that the ratio of rRNA to mRNA is constant as a standardized amount of extracted RNA is put in to any reverse transcription reaction. However, as 80% of the RNA in a cell is rRNA, any factor that reduces rRNA synthesis will alter the rRNA to mRNA ratio invalidating the central assumption that establishes housekeeping genes. We have used established housekeeping genes from other systems. Our data may therefore be limited by this assumption, but given the variability in muscle biopsy samples, we have used this accepted approach in the absence of a suitable alternative. In each experiment, variation was minimized by ensuring all samples in that analysis were reverse transcribed at the same time using the same master mix. This criticism is, however, relevant to all studies examining RNA expression in muscle samples of patients as it is not possible to assume that the mRNA to rRNA ratio is maintained. One alternative would be to spike in a known amount of an RNA that could account for the efficiencies of RNA extraction and reverse transcription. However, the small size and the nature of taking biopsies from humans determining the precise dry weight of tissue whilst retaining RNA integrity and caring for the safety of the individual preclude this from being a viable option.

Finally, the animal model that we have used (over‐expression following electroporation) causes a degree of muscle damage. It is therefore possible that we are looking at a specific effect of the miRNA on the background of repair rather than looking at the effects of the miRNA on normal physiology. We quantified repair by determining the proportion of centralized nuclei and found it to be low, suggesting that any effect was small. However, it should also be remembered that the muscle of patients with chronic diseases is not normal, and in patients with COPD who maintain their muscle mass, there is an increase in centralized myonuclei compared with controls, suggesting that there is an increased rate of myonuclear turnover.

## Conclusions

Our data show that miR‐424 targets the production of ribosomes and suppresses protein synthesis. Consistent with this observation, over‐expression of miR‐424 in muscle causes atrophy in an animal model. Finally, miR‐424 is elevated in patients with muscle wasting and is associated with the amount of muscle that patients will go on to lose following surgery. We therefore conclude that elevated miR‐424 contributes to the loss of muscle mass that occurs in COPD, in sarcopenia, and in patients in the ICU.

## Conflict of Interest

P.K. reports personal fees from GlaxoSmithKline (GSK), outside the submitted work; M.I.P. reports personal fees from GSK and grants and personal fees from Novartis, outside the submitted work; M.G. reports grants, personal fees, and non‐financial support from GSK; personal fees from BI; personal fees from Silence Therapeutics; and personal fees from Cell Catapult, outside the submitted work; all other authors have no conflicts of interest.

## Ethical approvals

Each clinical study was approved by the appropriate ethics committee. The appropriate study numbers are COPD, 06/Q0404/35 and 06/Q0410/54; ICUAW, 10/H0722/9; Sarcopenia, 07/Q0204/68; and aortic surgery, 07/Q0204/68. Written informed consent was obtained from all participants in the studies. The animal study was approved by the Imperial College Ethical Review Process and licensed by the UK Secretary of State for the Home Office under Project License PPL 70/8297.

## Author contributions

The overall study was designed by P.K. with contributions from M.G. and M.I.P. Patients were recruited, and samples were collected by R.P., S.A.N., S.B., A.A.S., and H.P. The laboratory studies were performed by M.C., R.F.G., R.P., J.P.L., and J.Y.L. P.K. wrote the first draft of the paper, and all authors provided critical appraisal and input into the manuscript.

## Supporting information


**Figure S1**: Quantification of UBTF levels in transfected cells
**Figure S2**: Puromycin quantification by Western blot
**Figure S3**: EGFP and centralised nuclei in the electroporated mouse muscle
**Figure S4**: miR‐424‐5p is associated with the expression of miR‐542‐5p and 3p in COPD muscle
**Figure S5**: Quantification of normaliser genes
**Table S1**: Primers used in this study
**Table S2**: Physiological characteristics of the COPD cohort
**Table S3**: Physiological characteristics of HSS cohort
**Table S4**: Physiological characteristics of the ICUAW cohort
**Table S5**: Physiological characteristics of aortic surgery patients
**Table S6**: Selected Predicted gene targets of miR‐424‐5p

## References

[1] Takada H , Kurisaki A . Emerging roles of nucleolar and ribosomal proteins in cancer, development, and aging. Cell Mol Life Sci 2015;72:4015–4025. 26206377 10.1007/s00018-015-1984-1 PMC11113460

[2] Glass DJ . Skeletal muscle hypertrophy and atrophy signaling pathways. Int J Biochem Cell Biol 2005;37:1974–1984. 16087388 10.1016/j.biocel.2005.04.018

[3] Gordon BS , Kelleher AR , Kimball SR . Regulation of muscle protein synthesis and the effects of catabolic states. Int J Biochem Cell Biol 2013;45:2147–2157. 23769967 10.1016/j.biocel.2013.05.039 PMC3759561

[4] Lodish HF , Berk A , Zipursky SL , Matsudaira P , Baltimore D , Darnell J . Section 11.6 Processing of rRNA and tRNA In Molecular Cell Biology, 4th edition New York: Freeman W.H; 2000.

[5] Nazar RN . Ribosomal RNA processing and ribosome biogenesis in eukaryotes. IUBMB Life 2004;56:457–465. 15545225 10.1080/15216540400010867

[6] Hoppe S , Bierhoff H , Cado I , Weber A , Tiebe M , Grummt I , et al. AMP‐activated protein kinase adapts rRNA synthesis to cellular energy supply. Proc Natl Acad Sci U S A 2009;106:17781–17786. 19815529 10.1073/pnas.0909873106 PMC2764937

[7] Thomas G . An encore for ribosome biogenesis in the control of cell proliferation. Nat Cell Biol 2000;2:E71–E72. 10806485 10.1038/35010581

[8] Wolfe RR . The underappreciated role of muscle in health and disease. Am J Clin Nutr 2006;84:475–482. 16960159 10.1093/ajcn/84.3.475

[9] Gore DC , Jahoor F . Deficiency in peripheral glutamine production in pediatric patients with burns. J Burn Care Rehabil 2000;21:171, discussion 2‐7. 10752751 10.1097/00004630-200021020-00017

[10] Lightfoot A , McArdle A , Griffiths RD . Muscle in defense. Crit Care Med 2009;37:S384–S390. 20046124 10.1097/CCM.0b013e3181b6f8a5

[11] Biolo G , Fleming RY , Maggi SP , Nguyen TT , Herndon DN , Wolfe RR . Inhibition of muscle glutamine formation in hypercatabolic patients. Clin Sci (Lond) 2000;99:189–194. 11787470

[12] Cruzat VF , Krause M , Newsholme P . Amino acid supplementation and impact on immune function in the context of exercise. J Int Soc Sports Nutr 2014;11:61. 25530736 10.1186/s12970-014-0061-8 PMC4272512

[13] Londhe P , Guttridge DC . Inflammation induced loss of skeletal muscle. Bone 2015;80:131–142. 26453502 10.1016/j.bone.2015.03.015 PMC4600538

[14] Toth MJ , LeWinter MM , Ades PA , Matthews DE . Impaired muscle protein anabolic response to insulin and amino acids in heart failure patients: relationship with markers of immune activation. Clin Sci (Lond) 2010;119:467–476. 20528773 10.1042/CS20100110 PMC3032598

[15] Rennie MJ . Anabolic resistance in critically ill patients. Crit Care Med 2009;37:S398–S399. 20046126 10.1097/CCM.0b013e3181b6ec1f

[16] Cuthbertson D , Smith K , Babraj J , Leese G , Waddell T , Atherton P , et al. Anabolic signaling deficits underlie amino acid resistance of wasting, aging muscle. FASEB J 2005;19:422–424. 15596483 10.1096/fj.04-2640fje

[17] Cicoira M , Kalra PR , Anker SD . Growth hormone resistance in chronic heart failure and its therapeutic implications. J Card Fail 2003;9:219–226. 12815572 10.1054/jcaf.2003.23

[18] Guillet C , Prod'homme M , Balage M , Gachon P , Giraudet C , Morin L , et al. Impaired anabolic response of muscle protein synthesis is associated with S6K1 dysregulation in elderly humans. FASEB J 2004;18:1586–1587. 15319361 10.1096/fj.03-1341fje

[19] Pocock SJ , McMurray JJ , Dobson J , Yusuf S , Granger CB , Michelson EL , et al. Weight loss and mortality risk in patients with chronic heart failure in the candesartan in heart failure: assessment of reduction in mortality and morbidity (CHARM) programme. Eur Heart J 2008;29:2641–2650. 18819960 10.1093/eurheartj/ehn420

[20] Schols AM , Broekhuizen R , Weling‐Scheepers CA , Wouters EF . Body composition and mortality in chronic obstructive pulmonary disease. Am J Clin Nutr 2005;82:53–59. 16002800 10.1093/ajcn.82.1.53

[21] Swallow EB , Reyes D , Hopkinson NS , Man WD , Porcher R , Cetti EJ , et al. Quadriceps strength predicts mortality in patients with moderate to severe chronic obstructive pulmonary disease. Thorax 2007;62:115–120. 17090575 10.1136/thx.2006.062026 PMC2111256

[22] He L , Hannon GJ . MicroRNAs: small RNAs with a big role in gene regulation. Nat Rev Genet 2004;5:522–531. 15211354 10.1038/nrg1379

[23] Rivas DA , Lessard SJ , Rice NP , Lustgarten MS , So K , Goodyear LJ , et al. Diminished skeletal muscle microRNA expression with aging is associated with attenuated muscle plasticity and inhibition of IGF‐1 signaling. FASEB J 2014;28:4133–4147. 24928197 10.1096/fj.14-254490 PMC5058318

[24] Wang Y , Huang JW , Castella M , Huntsman DG , Taniguchi T . p53 is positively regulated by miR‐542‐3p. Cancer Res 2014;74:3218–3227. 24762395 10.1158/0008-5472.CAN-13-1706 PMC4058365

[25] Orom UA , Nielsen FC , Lund AH . MicroRNA‐10a binds the 5′UTR of ribosomal protein mRNAs and enhances their translation. Mol Cell 2008;30:460–471. 18498749 10.1016/j.molcel.2008.05.001

[26] Farre Garros R , Paul R , Connolly M , Lewis A , Garfield BE , Natanek SA , et al. miR‐542 promotes mitochondrial dysfunction and SMAD activity and is raised in ICU acquired weakness. Am J Respir Crit Care Med 2017;https://doi.org/10.1164/rccm.201701-0101OC. 10.1164/rccm.201701-0101OC PMC5736972 28809518

[27] Zhu CH , Mouly V , Cooper RN , Mamchaoui K , Bigot A , Shay JW , et al. Cellular senescence in human myoblasts is overcome by human telomerase reverse transcriptase and cyclin‐dependent kinase 4: consequences in aging muscle and therapeutic strategies for muscular dystrophies. Aging Cell 2007;6:515–523. 17559502 10.1111/j.1474-9726.2007.00306.x

[28] Martin KM , Cooper WN , Metcalfe JC , Kemp PR . Mouse BTEB3, a new member of the basic transcription element binding protein (BTEB) family, activates expression from GC‐rich minimal promoter regions. Biochem J 2000;345:529–533. 10642511 PMC1220787

[29] Lewis A , Riddoch‐Contreras J , Natanek SA , Donaldson A , Man WD , Moxham J , et al. Downregulation of the serum response factor/miR‐1 axis in the quadriceps of patients with COPD. Thorax 2012;67:26–34. 21998125 10.1136/thoraxjnl-2011-200309 PMC3240776

[30] Donaldson A , Natanek SA , Lewis A , Man WD , Hopkinson NS , Polkey MI , et al. Increased skeletal muscle‐specific microRNA in the blood of patients with COPD. Thorax 2013;68:1140–1149. 23814167 10.1136/thoraxjnl-2012-203129 PMC3841809

[31] Lewis A , Lee JY , Donaldson AV , Natanek SA , Vaidyanathan S , Man WD , et al. Increased expression of H19/miR‐675 is associated with a low fat‐free mass index in patients with COPD. J Cachexia Sarcopenia Muscle 2016;7:330–344. 27239417 10.1002/jcsm.12078 PMC4863928

[32] Ellis PD , Smith CW , Kemp P . Regulated tissue‐specific alternative splicing of enhanced green fluorescent protein transgenes conferred by alpha‐tropomyosin regulatory elements in transgenic mice. J Biol Chem 2004;279:36660–36669. 15194683 10.1074/jbc.M405380200

[33] Gollins H , McMahon J , Wells KE , Wells DJ . High‐efficiency plasmid gene transfer into dystrophic muscle. Gene Ther 2003;10:504–512. 12621454 10.1038/sj.gt.3301927

[34] McMahon JM , Signori E , Wells KE , Fazio VM , Wells DJ . Optimisation of electrotransfer of plasmid into skeletal muscle by pretreatment with hyaluronidase—increased expression with reduced muscle damage. Gene Ther 2001;8:1264–1270. 11509960 10.1038/sj.gt.3301522

[35] Natanek SA , Gosker HR , Slot IG , Marsh GS , Hopkinson NS , Man WD , et al. Heterogeneity of quadriceps muscle phenotype in chronic obstructive pulmonary disease (COPD); implications for stratified medicine? Muscle Nerve 2013;48:488–497. 23553751 10.1002/mus.23784

[36] ATS statement: guidelines for the six‐minute walk test. Am J Respir Crit Care Med 2002;166:111–117. 12091180 10.1164/ajrccm.166.1.at1102

[37] Bergstrom J . Percutaneous needle biopsy of skeletal muscle in physiological and clinical research. Scand J Clin Lab Invest 1975;35:609–616. 1108172

[38] Patel HP , Jameson KA , Syddall HE , Martin HJ , Stewart CE , Cooper C , et al. Developmental influences, muscle morphology, and sarcopenia in community‐dwelling older men. J Gerontol A Biol Sci Med Sci 2012;67:82–87. 21357193 10.1093/gerona/glr020

[39] Patel H , Syddall HE , Martin HJ , Cooper C , Stewart C , Sayer AA . The feasibility and acceptability of muscle biopsy in epidemiological studies: findings from the Hertfordshire Sarcopenia Study (HSS). J Nutr Health Aging 2011;15:10–15. 21267515 10.1007/s12603-011-0006-8 PMC12879697

[40] Bloch SA , Lee JY , Syburra T , Rosendahl U , Griffiths MJ , Kemp PR , et al. Increased expression of GDF‐15 may mediate ICU‐acquired weakness by down‐regulating muscle microRNAs. Thorax 2015;70:219–228. 25516419 10.1136/thoraxjnl-2014-206225 PMC4345798

[41] Seymour JM , Ward K , Sidhu PS , Puthucheary Z , Steier J , Jolley CJ , et al. Ultrasound measurement of rectus femoris cross‐sectional area and the relationship with quadriceps strength in COPD. Thorax 2009;64:418–423. 19158125 10.1136/thx.2008.103986

[42] Dweep H , Sticht C , Pandey P , Gretz N . miRWalk—database: prediction of possible miRNA binding sites by “walking” the genes of three genomes. J Biomed Inform 2011;44:839–847. 21605702 10.1016/j.jbi.2011.05.002

[43] Xiao X , Huang C , Zhao C , Gou X , Senavirathna LK , Hinsdale M , et al. Regulation of myofibroblast differentiation by miR‐424 during epithelial‐to‐mesenchymal transition. Arch Biochem Biophys 2015;566:49–57. 25524739 10.1016/j.abb.2014.12.007 PMC4297572

[44] Llobet‐Navas D , Rodriguez‐Barrueco R , de la Iglesia‐Vicente J , Olivan M , Castro V , Saucedo‐Cuevas L , et al. The microRNA 424/503 cluster reduces CDC25A expression during cell cycle arrest imposed by transforming growth factor beta in mammary epithelial cells. Mol Cell Biol 2014;34:4216–4231. 25266660 10.1128/MCB.00611-14 PMC4248740

[45] Marchand A , Atassi F , Mougenot N , Clergue M , Codoni V , Berthuin J , et al. miR‐322 regulates insulin signaling pathway and protects against metabolic syndrome‐induced cardiac dysfunction in mice. Biochim Biophys Acta 2016;1862:611–621. 26775030 10.1016/j.bbadis.2016.01.010

[46] Wang F , Wang J , Yang X , Chen D , Wang L . miR‐424‐5p participates in esophageal squamous cell carcinoma invasion and metastasis via SMAD7 pathway mediated EMT. Diagn Pathol 2016;11:88. 27628042 10.1186/s13000-016-0536-9 PMC5024440

[47] Nakada S , Ogasawara R , Kawada S , Maekawa T , Ishii N . Correlation between ribosome biogenesis and the magnitude of hypertrophy in overloaded skeletal muscle. PLoS One 2016;11:e0147284. 26824605 10.1371/journal.pone.0147284 PMC4732984

[48] Goodman CA , Frey JW , Mabrey DM , Jacobs BL , Lincoln HC , You JS , et al. The role of skeletal muscle mTOR in the regulation of mechanical load‐induced growth. J Physiol 2011;589:5485–5501. 21946849 10.1113/jphysiol.2011.218255 PMC3240886

[49] von Walden F , Casagrande V , Ostlund Farrants AK , Nader GA . Mechanical loading induces the expression of a Pol I regulon at the onset of skeletal muscle hypertrophy. Am J Physiol Cell Physiol 2012;302:C1523–C1530. 22403788 10.1152/ajpcell.00460.2011

[50] Chaillou T , Lee JD , England JH , Esser KA , McCarthy JJ . Time course of gene expression during mouse skeletal muscle hypertrophy. J Appl Physiol 2013;115:1065–1074. 23869057 10.1152/japplphysiol.00611.2013 PMC3798821

[51] von Walden F , Liu C , Aurigemma N , Nader GA . mTOR signaling regulates myotube hypertrophy by modulating protein synthesis, rDNA transcription, and chromatin remodeling. Am J Physiol Cell Physiol 2016;311:C663–CC72. 27581648 10.1152/ajpcell.00144.2016

[52] Hannan KM , Brandenburger Y , Jenkins A , Sharkey K , Cavanaugh A , Rothblum L , et al. mTOR‐dependent regulation of ribosomal gene transcription requires S6K1 and is mediated by phosphorylation of the carboxy‐terminal activation domain of the nucleolar transcription factor UBF. Mol Cell Biol 2003;23:8862–8877. 14612424 10.1128/MCB.23.23.8862-8877.2003 PMC262650

[53] Figueiredo VC , Caldow MK , Massie V , Markworth JF , Cameron‐Smith D , Blazevich AJ . Ribosome biogenesis adaptation in resistance training‐induced human skeletal muscle hypertrophy. Am J Physiol Endocrinol Metab 2015;309:E72–E83. 25968575 10.1152/ajpendo.00050.2015

[54] Machida M , Takeda K , Yokono H , Ikemune S , Taniguchi Y , Kiyosawa H , et al. Reduction of ribosome biogenesis with activation of the mTOR pathway in denervated atrophic muscle. J Cell Physiol 2012;227:1569–1576. 21678406 10.1002/jcp.22871

[55] Larson DE , Xie W , Glibetic M , O'Mahony D , Sells BH , Rothblum LI . Coordinated decreases in rRNA gene transcription factors and rRNA synthesis during muscle cell differentiation. Proc Natl Acad Sci U S A 1993;90:7933–7936. 8396256 10.1073/pnas.90.17.7933 PMC47261

[56] Jorgensen P , Rupes I , Sharom JR , Schneper L , Broach JR , Tyers M . A dynamic transcriptional network communicates growth potential to ribosome synthesis and critical cell size. Genes Dev 2004;18:2491–2505. 15466158 10.1101/gad.1228804 PMC529537

[57] Boulon S , Westman BJ , Hutten S , Boisvert FM , Lamond AI . The nucleolus under stress. Mol Cell 2010;40:216–227. 20965417 10.1016/j.molcel.2010.09.024 PMC2987465

[58] Olson MO . Sensing cellular stress: another new function for the nucleolus? Sci STKE 2004;2004:pe10. 15026578 10.1126/stke.2242004pe10

[59] Jaitovich A , Angulo M , Lecuona E , Dada LA , Welch LC , Cheng Y , et al. High CO_2_ levels cause skeletal muscle atrophy via AMP‐activated kinase (AMPK), FoxO3a protein, and muscle‐specific RING finger protein 1 (MuRF1). J Biol Chem 2015;290:9183–9194. 25691571 10.1074/jbc.M114.625715 PMC4423704

[60] Rivas DA , McDonald DJ , Rice NP , Haran PH , Dolnikowski GG , Fielding RA . Diminished anabolic signaling response to insulin induced by intramuscular lipid accumulation is associated with inflammation in aging but not obesity. Am J Physiol Regul Integr Comp Physiol 2016;310:R561–R569. 26764052 10.1152/ajpregu.00198.2015 PMC4867383

[61] Lang CH , Frost RA , Vary TC . Regulation of muscle protein synthesis during sepsis and inflammation. Am J Physiol Endocrinol Metab 2007;293:E453–E459. 17505052 10.1152/ajpendo.00204.2007

[62] Haran PH , Rivas DA , Fielding RA . Role and potential mechanisms of anabolic resistance in sarcopenia. J Cachexia Sarcopenia Muscle 2012;3:157–162. 22589021 10.1007/s13539-012-0068-4 PMC3424190

[63] Clowes GH Jr , Randall HT , Cha CJ . Amino acid and energy metabolism in septic and traumatized patients. JPEN J Parenter Enteral Nutr 1980;4:195–205. 6995629 10.1177/014860718000400225

[64] Vesali RF , Klaude M , Rooyackers OE , TJäder I , Barle H , Wernerman J . Longitudinal pattern of glutamine/glutamate balance across the leg in long‐stay intensive care unit patients. Clin Nutr 2002;21:505–514. 12468371 10.1054/clnu.2002.0583

[65] McPherron AC , Lawler AM , Lee SJ . Regulation of skeletal muscle mass in mice by a new TGF‐beta superfamily member. Nature 1997;387:83–90. 9139826 10.1038/387083a0

[66] Lee JY , Donaldson AV , Lewis A , Natanek SA , Polkey MI , Kemp PR . Circulating miRNAs from imprinted genomic regions are associated with peripheral muscle strength in COPD patients. Eur Respir J 2017;49:1601881; https://doi.org/10.1183/13993003.01881-2016. 10.1183/13993003.01881-2016 28446557

[67] Lewis A , Lee JY , Donaldson AV , Natanek SA , Vaidyanathan S , Man WD , et al. Increased expression of H19/miR‐675 is associated with a low fat free mass index in patients with COPD. J Sarcopenia Cachexia and Muscle 2016;7:330–344. 10.1002/jcsm.12078 PMC4863928 27239417

[68] von Haehling S , Morley JE , Coats AJ , Anker SD . Ethical guidelines for publishing in the Journal of Cachexia, Sarcopenia and Muscle: update 2015. J Cachexia Sarcopenia Muscle 2015;6:315–316. 26672494 10.1002/jcsm.12089 PMC4670739

