# Supplementary material for: miR‐424‐5p reduces ribosomal RNA and protein synthesis in muscle wasting
Source: J Cachexia Sarcopenia Muscle. 2017 Dec 7;9(2):400–16. doi: 10.1002/jcsm.12266 (PMC5879973; doi:10.1002/jcsm.12266)
Supplement: Supplementary file 1 — Figure S1: Quantification of UBTF levels in transfected cells Figure S2: Puromycin quantification by Western blot Figure S3: EGFP and centralised nuclei in the electroporated mouse muscle Figure S4: miR‐424‐5p is associated with the expression of miR‐542‐5p and 3p in COPD muscle Figure S5: Quantification of normaliser genes Table S1: Primers used in this study Table S2: Physiological characteristics of the COPD cohort Table S3: Physiological characteristics of HSS cohort Table S4: Physiological characteristics of the ICUAW cohort Table S5: Physiological characteristics of aortic surgery patients Table S6: Selected Predicted gene targets of miR‐424‐5p [file JCSM-9-400-s001.zip › 424 SUPPLEMENTARY Information revised.docx]

**Supplementary Results**

**RNA quantification from *in vitro* experiments**

**18S rRNA is commonly used as a housekeeping gene, however in this series of experiments we identify a reduction in the expression of this gene relative to the normalisers that we use. Consequently, it is possible that we are observing a change in the normalisers rather than the 18S rRNA. We therefore analysed the levels of the normaliser genes used to ensure that the levels of these genes did not change significantly with each treatment. CT values for both B2M and GAPDH did not vary across the data set (424 vs control, 424 vs [antagomiR + 424] or [antagomiR + 424] vs control) either in each of the 4 experiments considered alone or in all 4 experiments combined (Fig S5). The Geomean of these values and the normalised geomean of the values was also consistent and did not vary (Fig. S5). The normalisers chosen does not therefore appear to have driven the differences in 18S rRNA quantification.**

**Supplementary Figure Legends**

**Figure S1. Quantification of UBTF levels in transfected cells**

**Protein extracted from LHCN-M2 cells 48h after transfection with miR-424 (424), miR-424 and the antagomiR (424+A) or control miRNA (cont) was analysed by Western blot for UBTF as described in Methods. Blots were scanned and the images analysed in Fiji. Raw UBTF levels and Ponceau S levels for all samples are shown in (A) and the normalised data shown in (B). In each experiment 3 independent transfections were performed and the experiment was three times. To reduce inter-experiment variation the data from each sample was normalised to the average of the control values for the corresponding experiment. One data point from was removed due to poor transfer of that particular sample (as shown by its Ponceau S stain). The box and whisker plots show median and interquartile range with the whiskers to the 90^th^ centiles and outliers shown.**

**Figure S2: Puromycin quantification by Western blot**

**14 samples used to quantify protein synthesis (7 transfected with miR-424 mimic and 7 with control miRNA) for which there was 5μg of protein left after ELISA were electrophoresed through 4-12% polyacrylamide gels and puromycin incorporation was quantified by Western blotting (A). Chemiluminescent signal from each lane was quantified and normalised to Ponceau S staining for the same lane. Transfection of the cells with miR-424-5p reduced protein synthesis compared to transfection with a control miRNA (B). The box and whisker plots show median and interquartile range with the whiskers to the 90^th^ centiles and outliers shown.**

**Figure S3 EGFP and centralised nuclei in the electroporated mouse muscle**

**Adjacent sections from the TAs of mice electroporated with pCAGGS-EGFP-424 into one leg and pCAGGS-EGFP into the contralateral limb were stained with Hemotoxylin and Eosin and imaged under bright field or imaged for EGFP fluorescence. The proportion of fibres with centralised nuclei was determined in the H & E stained sections and the electroporation efficiency was determined in the fluorescence images. In the regions imaged only 2% of the fibres contained centralised nuclei and there was no difference between the control and miR-424 transfected muscles (A). There was also no difference in the proportion of fibres expressing EGFP (B). (C) Representative H&E images and the corresponding fluorescence images are shown. The box and whisker plots show median and interquartile range with the whiskers to the 90^th^ centiles and outliers shown.**

**Figure S4 miR-424-5p is associated with the expression of miR-542-5p and 3p in COPD muscle**

Comparison of the expression of miR-424-5p with miR-542-3p(A) and miR-542-5p (B) **in quadriceps biopsies from COPD patients** showed tight correlations.

**Figure S5 Quantification of normaliser genes**

**CT values for the individual normaliser genes and the geomean of the two values are shown for cells transfected with miR-424 (424), miR-424 and the antagomiR (424+A) or control miRNA (cont) (A). These data were derived from 4 independent experiments of n=6. The Geomean was normalised to remove any inter-experiment variation by normalising each value to the average of the control values for that experiment (B). The data are presented as box plots with whiskers to the 90^th^ centile with outliers shown.**

**SUPPLEMENTARY TABLES**

**Table S1 Primers used in this study**

| **Primer** | **Species** | **Forward Seq.** | **Reverse Seq.** |
| --- | --- | --- | --- |
| 18S | Human | GTAACCCGTTGAACCCCATT | CCATCCAATCGGTAGTAGCG |
| 28S | Human | ACGGCGGGAGTAACTCTGACT | CTTGGCTGTGGTTTCGCT |
| 47S / Pre-45S | Human | CCTTCCCCAGGCGTCCCTCG | GGCAGCGCTACCATAACGGA |
| GAPDH | Human | GGTGGTCTCCTCTGACTTCAACA | GTTGCTGTAGCCAAATTCGTTGT |
| PolR1A | Human | TCCCATGGTGTTTGCCACAA | TGCACATTAGGGCCGTTGAT |
| RRN3 | Human | CCAGACATAAAGGATGACCAGAT | GCTCAAAGTCTTTTGTCAAGTACA |
| β2 Microglobulin | Human | TGCTGTCTCCATGTTTGATGTATCT | TCTCTGCTCCCCACCTCTAAGT |
| 28S | Mouse | TGCCATGGTAATCCTGCTCA | CCTCAGCCAAGCACATACACC |
| 47S | Mouse | CCAAGTGTTCATGCCACGTG | CGAGCGACTGCCACAAAAA |
| BMPR1A | Mouse | CAGACTTGGACCAGAAGAAGCC | ACATTCTATTGTCCTGCGTAGC |
| BMPR2 | Mouse | AGGCCCAATTCTCTGGATCT | CACTGCCATTGTTGTTGACC |
| CDC25A | Mouse | AGAACCCTATTGTGCCTACTG | TACTCATTGCCGAGCCTATC |
| DiO3 | Mouse | TTTGGTCTCGAAGTCCATCC | GCCTCTAACTGGGCTTGACC |
| IGF-1 | Mouse | CACCTCAGACAGGCATTGTG | TCTGAGTCTTGGGCATGTCA |
| IGF-2 | Mouse | GTGCTGCATCGCTGCTTAC | ACGTCCCTCTCGGACTTGG |
| IGF-2R | Mouse | GGGAAGCTGTTGACTCCAAAA | GCAGCCCATAGTGGTGTTGAA |
| NfkB | Mouse | ATCTTCACCATGGCAGACGAT | TGAGTGAGTCAAAGCAGTGTTCAA |
| P70S6K | Mouse | GGAGTTTGGGAGCATTAATGTATG | GGTAGGGAGGCAAATTAAGTTTAC |
| PolR1A | Mouse | CCCACTGTGGACAGGAAAA | GGAGTCCGGGTCAAAGT |
| SMAD1 | Mouse | GTGGAAACAGGGCGATGAAG | GCGTCCACAGCTTTCTCTGC |
| SMAD7 | Mouse | TCGGACAGCTCAATTCGGAC | GGTAACTGCTGCGGTTGTAA |
| Sox 6 | Mouse | TGCGACAGTTCTTCACTGTGG | CGTCCATCTTCATACCATACG |
| SURVIVIN | Mouse | AGAACTGGCCCTTCTTGGAGG | CTTTTTATGTTCCTCTATGGGGT |
| TFAM | Mouse | CTGATGGGTATGGAGAAGGAGG | CCAACTTCAGCCATCTGCTCTTC |
| UBTF | Mouse | GTCGGACATCCCCGAGAAAC | GAGAGCTGAGACCACTGCTT |
| UCP3 | Mouse | GGAGTCTCACCTGTTTACTGACAACT | GCACAGAAGCCAGCTCCAA |
| β2 Microglobulin | Mouse | CCGTCTACTGGGATCGAGAC | GCTATTTCTTTCTGCGTGCAT |

**Table S2: Physiological characteristics of the COPD cohort**

|  | Control (n=16) | COPD (n=**49**) |
| --- | --- | --- |
| Sex | 6M/10F | 29M/20F |
| Age (years) | 65 ± 8 | 66 ± 8 |
| Smoking History^a^ (pack-year) | 0 (0,10) | 44 (30,60)*** |
| Weight ^a^ (kg) | 65 (61,74) | 67 (59, 77) |
| BMI^a^ (kg/m^2^) | 24.8 (23.5, 26.72) | 23.7 (21.7, 26.3) |
| FFMI ^a^ (kg/m^2^) | 16.0 (15.2, 16.9) | 15.4 (14.5, 16.8) |
| FEV_1_^a^ (% pred) | 106.8 (100.5, 111.5) | 40.7 (27.2, 48.4 )*** |
| TLCO ^a^ (% pred) | 87.5 (80.8, 98.1) | 45.0 (32.1,52.4 )*** |
| 6min walk (% pred) | 128 (123, 130) | 81 (63, 91)*** |
| SGRQ ^a^ | 2 (0, 7.5) | 54 (45, 62)*** |
| Quadriceps MVC (kg) | 34.1 (27.8, 37.2) | 27.2 (22.3, 34.2) |
| Quadriceps MVC (% pred) | 78±19 | 64 ± 14*** |
| Locomotion time^a^ (min/12 hr) | 97 (84, 128) | 42 (24, 62)*** |

BMI (Body mass index), FFMI (fat free mass index), FEV_1_ (Forced expiratory volume in 1 sec), RVTLC (ratio of the reserve volume to total lung capacity), TLCO (transfer capacity of the lung for CO) SGRQ (St George’s respiratory questionnaire) MVC (maximal voluntary contraction). * p<0.05, ** p<0.01, ***p<0.001. Data are presented as mean ±SD for normally distributed data or as median (interquartile range) for data that was not normally distributed

**Table S3 Physiological characteristics of HSS cohort**

|  | Non-sarcopenic (n=59) | Sarcopenic (n=5) |
| --- | --- | --- |
| Weight ^a^ (kg) | 83 (72, 92) | 69(66, 71)* |
| BMI^a^ (kg/m^2^) | 27 (25, 29) | 26 (25, 27) |
| FFMI ^a^ (kg/m^2^) | 18.5 (17.5, 19.3) | 16.2 (15.9, 17.1)** |
| FEV_1_^a^ (% pred) | 106 (98, 117) | 108 (103, 111) |
| TUG time (s) | 10.4 ± 1.7 | 13.7 ± 4.7** |
| 3m walk time ^a^ (s) | 2.7 ± .4 | 3.4 ± 1.0** |

BMI (Body mass index), FFMI (fat free mass index), FEV_1_ (Forced expiratory volume in 1 sec), TUG time (time to ‘get up and go’). ). * p<0.05, ** p<0.01. Data are presented as mean ±SD for normally distributed data or as median (interquartile range) for data that was not normally distributed.

**Table S4 Physiological characteristics of the ICUAW cohort**

|  | **Control** | **ICUAW** |
| --- | --- | --- |
| Sex (M/F) | 7/0 | 13/4 |
| Age (yr) | 68 ± 11 | 63 ± 17 |
| BMI (kg/m^2^) | 25 ± 4 | 28 ± 3 |
| Days on ICU | n/a | 21 ± 7 |
| SOFA score at biopsy | n/a | 11 5 |
| Muscle layer thickness (cm) | 2.7 ± 0.7 (n=5) | 1.9 ± 0.6 (n=12)* |

BMI (Body mass index). * p<0.05. Data are presented as mean ±SD for normally distributed data or as median (interquartile range) for data that was not normally distributed.

**Table S5 Physiological characteristics of aortic surgery patients**

|  | Non-wasting Patients (n=19) | Wasting Patients (n=21) | P-value |
| --- | --- | --- | --- |
| **Demographic data** |  |  |  |
| Age (yr) | 58.7 ± 15.4 | 68.1 ± 14.9 | ***** |
| Sex (M/F) | 17/4 | 17/7 |  |
| BMI (kg/m2) | 27.1 ± 3.6 | 27.5 ± 6.9 | NS |
| EuroSCORE 2 | 2.0 (1.3-3.5) | 3.0 (1.4-9.6) | NS |
| Pre-operative LVEF (%) | 59.6 ± 9.1 | 56.0 ± 10.6 | NS |
| Pre-operative creatinine clearance (μmol/L) | 74.1 ± 16.3 | 68.0 ± 20.4 | NS |
| SPPB | 12 (12,12) | 10 (10-12) | ******* |
| WHO performance status |  |  |  |
| Pre-operative | 0 (0-1) | 1 (0-1) | ***** |
| Post-operative | 1 (0-2) | 2 (1-3) | ***** |
| **Operative data** |  |  |  |
| Total bypass time (mins) | 143.0 ± 49.0 | 142.1 ± 55.7 | NS |
| Total cross-clamp time (mins) | 103.6 ± 35.4 | 95.7 ± 33.2 | NS |
| **Critical care data** |  |  |  |
| ICU length of stay, days | 1.0 (1-2) | 3.0 (2-7) | ******* |
| Hospital length of stay, days | 8 (7-12) | 12 (9-23) | ***** |
| Mechanical ventilation (hours) | 16 (13-24) | 26 (19-99) | ****** |
| Vasopressor duration (hours) | 27 (15-48) | 46 (22-186) | ***** |
| **Muscle data** |  |  |  |
| RF_CSA_ day 0 | 6.7 ± 2.2 | 6.1 ± 1.6 | NS |
| RF_CSA_ day 7 | 6.6 ± 2.2 | 5.2 ± 2.6 | ***** |
| Change in QMVC (%) | -4.3 ± 10.1 | -15.0 ± 14.3 | ***** |

BMI (Body mass index), LVEF (left ventricular ejection fraction), SPPB (Short physical performance battery). * p<0.05, ** p<0.01, ***p<0.001. Data are presented as mean ±SD for normally distributed data or as median (interquartile range) for data that was not normally distributed

**Table S6: Selected Predicted gene targets of miR-424-5p**

| **Gene name** | **Protein** |
| --- | --- |
| **rRNA synthesis** | |
| PolR1A | DNA-directed RNA polymerase I subunit RPA1 |
| UBTF | Upstream binding transcription factor 1 |
| **Ribosome** |  |
| EIF1AX | Eukaryotic translation initiation factor 1A, X-chromosomal |
| EIF2S2 | Eukaryotic translation initiation factor 2 subunit 2 |
| EIF3A | Eukaryotic translation initiation factor 3 subunit A |
| EIF4B | Eukaryotic translation initiation factor 4B |
| EIF4E2 | Eukaryotic translation initiation factor 4E type 2 |
| EIF4G1 | Eukaryotic translation initiation factor 4 gamma 1 |
| EIF5A | Eukaryotic translation initiation factor 5A |
| EIF5B | Eukaryotic translation initiation factor 5B |
| RPS6KA3 | Ribosomal protein S6 kinase alpha-3 |
| RPS6KB1 | Ribosomal protein S6 kinase beta-1 |
| RPS14 | 40S ribosomal protein S14 |
| RPS9 | 40S ribosomal protein S9 |
| RPS23 | 40S ribosomal protein S23 |
| **TGF-b signalling inhibitors** | |
| SMURF1 | E3 ubiquitin-protein ligase SMURF1 |
| SMURF2 | E3 ubiquitin-protein ligase SMURF2 |
| SMAD7 | Mothers against decapentaplegic homolog 7 |
| **Insulin/IGF-1 signalling pathway** | |
| IGF-1 | insulin like growth factor |
| IGF1R | insulin like growth factor receptor 1 |
| INSR | insulin receptor |
| IRS1 | insulin receptor substrate 1(IRS1) |
| IRS2 | insulin receptor substrate 2(IRS2) |
| KRAS | GTPase(KRAS) |
| MAPK1 | mitogen-activated protein kinase 1(MAPK1) |
| MAPK3 | mitogen-activated protein kinase 3(MAPK3) |
| MAPK8 | mitogen-activated protein kinase 8(MAPK8) |
| MAPK9 | mitogen-activated protein kinase 9(MAPK9) |
| MAP2K1 | mitogen-activated protein kinase kinase 1(MAP2K1) |
| MAP2K2 | mitogen-activated protein kinase kinase 1(MAP2K2) |
| PIK3R1 | phosphoinositide-3-kinase regulatory subunit 1 |
| PIK3R2 | phosphoinositide-3-kinase regulatory subunit 2 |
| PIK3R3 | phosphoinositide-3-kinase regulatory subunit 3 |
| RAF1 | serine/threonine kinase(RAF1) |
| TSC1 | tuberous sclerosis 1(TSC1) |

Genes were selected as predicted by more than 5 miRNA target prediction algorithms in miRWalk2.0 and known to be associated with rRNA or protein synthesis or inhibitors of protein degradation.
