# Supplementary figures and images for: miR‐424‐5p reduces ribosomal RNA and protein synthesis in muscle wasting
Source: J Cachexia Sarcopenia Muscle. 2017 Dec 7;9(2):400–16. doi: 10.1002/jcsm.12266 (PMC5879973; doi:10.1002/jcsm.12266)

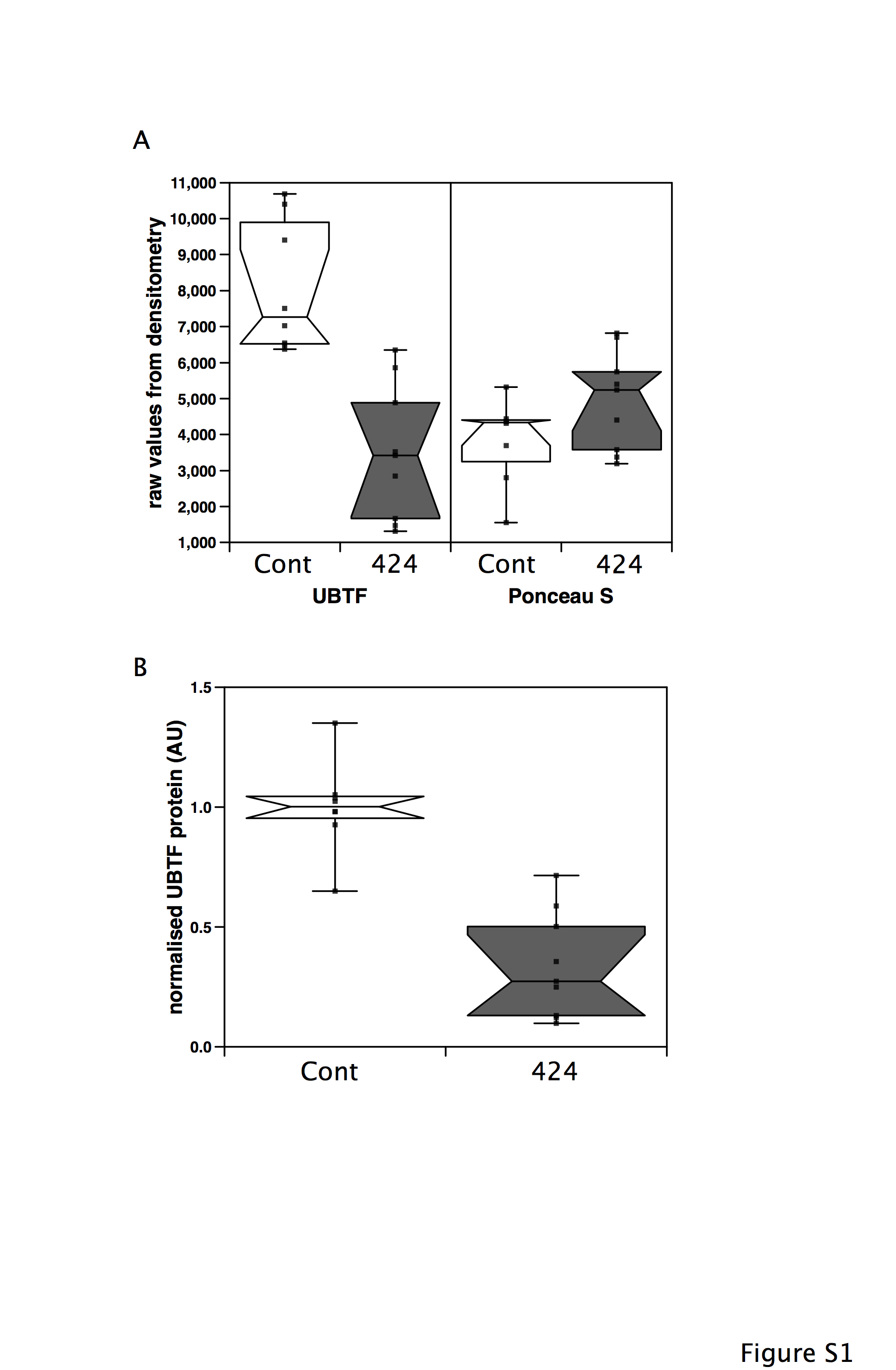

Supplement: Supplementary file 1 — Figure S1: Quantification of UBTF levels in transfected cells Figure S2: Puromycin quantification by Western blot Figure S3: EGFP and centralised nuclei in the electroporated mouse muscle Figure S4: miR‐424‐5p is associated with the expression of miR‐542‐5p and 3p in COPD muscle Figure S5: Quantification of normaliser genes Table S1: Primers used in this study Table S2: Physiological characteristics of the COPD cohort Table S3: Physiological characteristics of HSS cohort Table S4: Physiological characteristics of the ICUAW cohort Table S5: Physiological characteristics of aortic surgery patients Table S6: Selected Predicted gene targets of miR‐424‐5p [file JCSM-9-400-s001.zip › 424 and rRNA fig s1.tif]

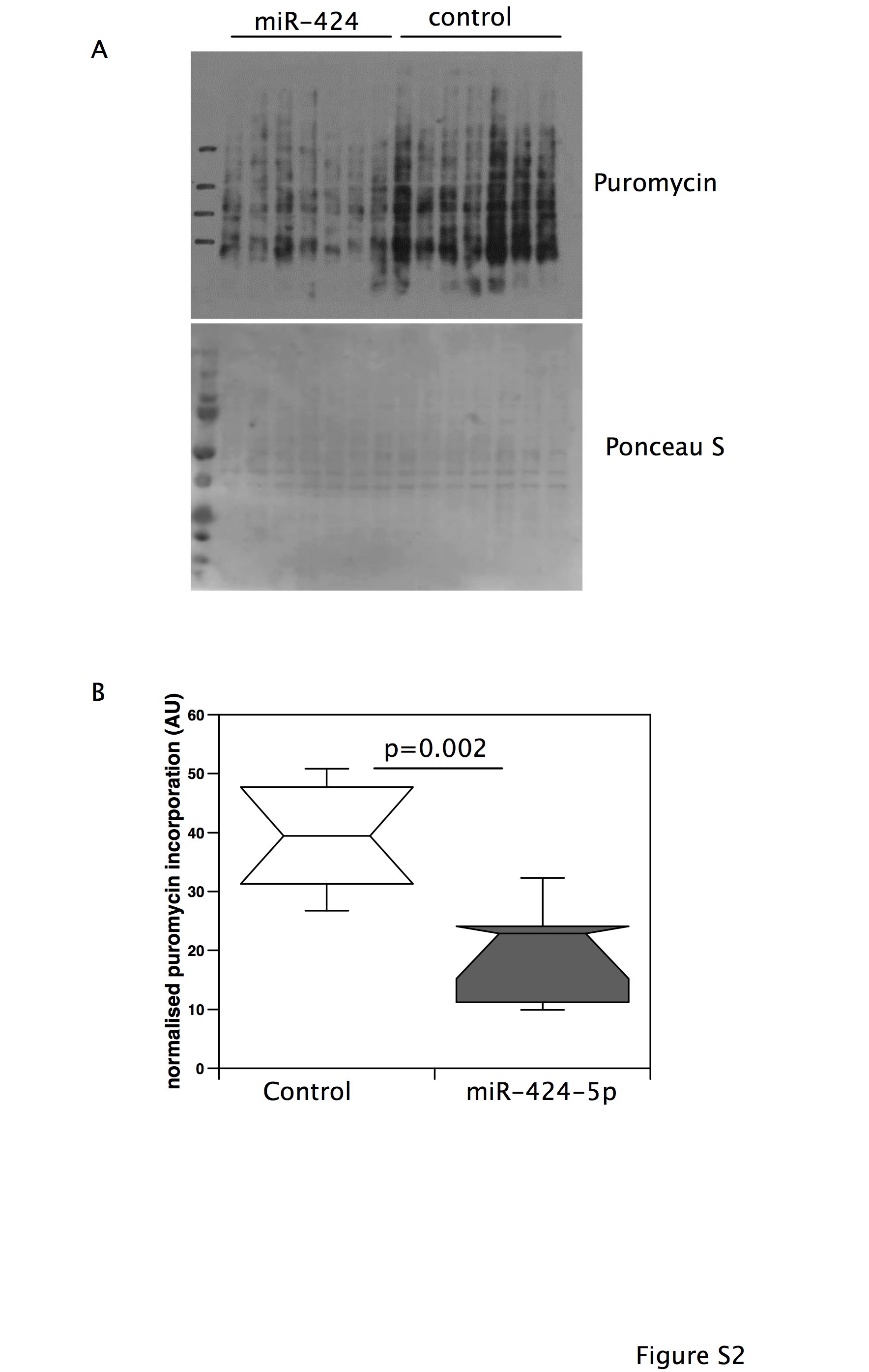

Supplement: Supplementary file 1 — Figure S1: Quantification of UBTF levels in transfected cells Figure S2: Puromycin quantification by Western blot Figure S3: EGFP and centralised nuclei in the electroporated mouse muscle Figure S4: miR‐424‐5p is associated with the expression of miR‐542‐5p and 3p in COPD muscle Figure S5: Quantification of normaliser genes Table S1: Primers used in this study Table S2: Physiological characteristics of the COPD cohort Table S3: Physiological characteristics of HSS cohort Table S4: Physiological characteristics of the ICUAW cohort Table S5: Physiological characteristics of aortic surgery patients Table S6: Selected Predicted gene targets of miR‐424‐5p [file JCSM-9-400-s001.zip › 424 and rRNA fig s2.tif]

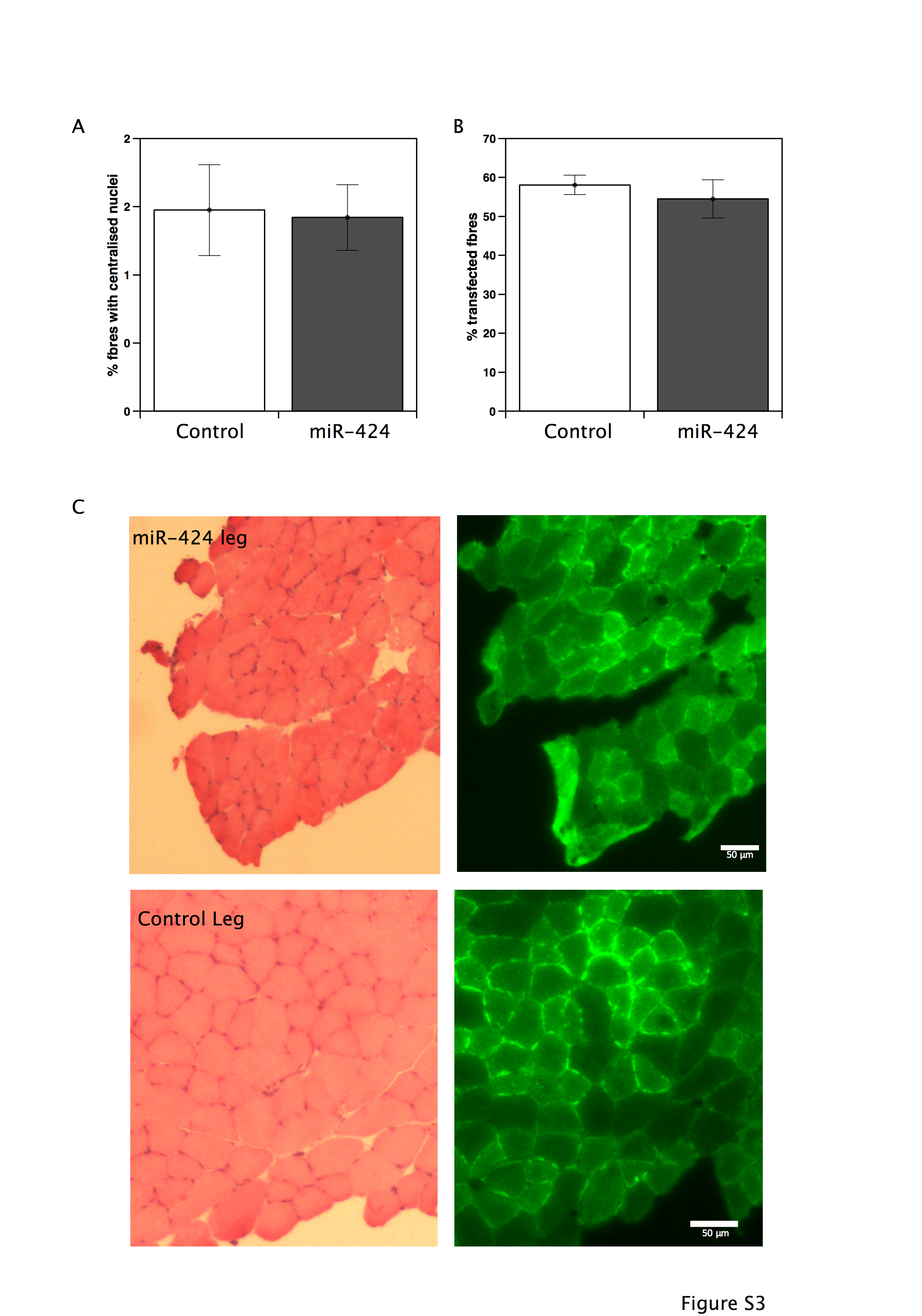

Supplement: Supplementary file 1 — Figure S1: Quantification of UBTF levels in transfected cells Figure S2: Puromycin quantification by Western blot Figure S3: EGFP and centralised nuclei in the electroporated mouse muscle Figure S4: miR‐424‐5p is associated with the expression of miR‐542‐5p and 3p in COPD muscle Figure S5: Quantification of normaliser genes Table S1: Primers used in this study Table S2: Physiological characteristics of the COPD cohort Table S3: Physiological characteristics of HSS cohort Table S4: Physiological characteristics of the ICUAW cohort Table S5: Physiological characteristics of aortic surgery patients Table S6: Selected Predicted gene targets of miR‐424‐5p [file JCSM-9-400-s001.zip › 424 and rRNA fig s3.tif]

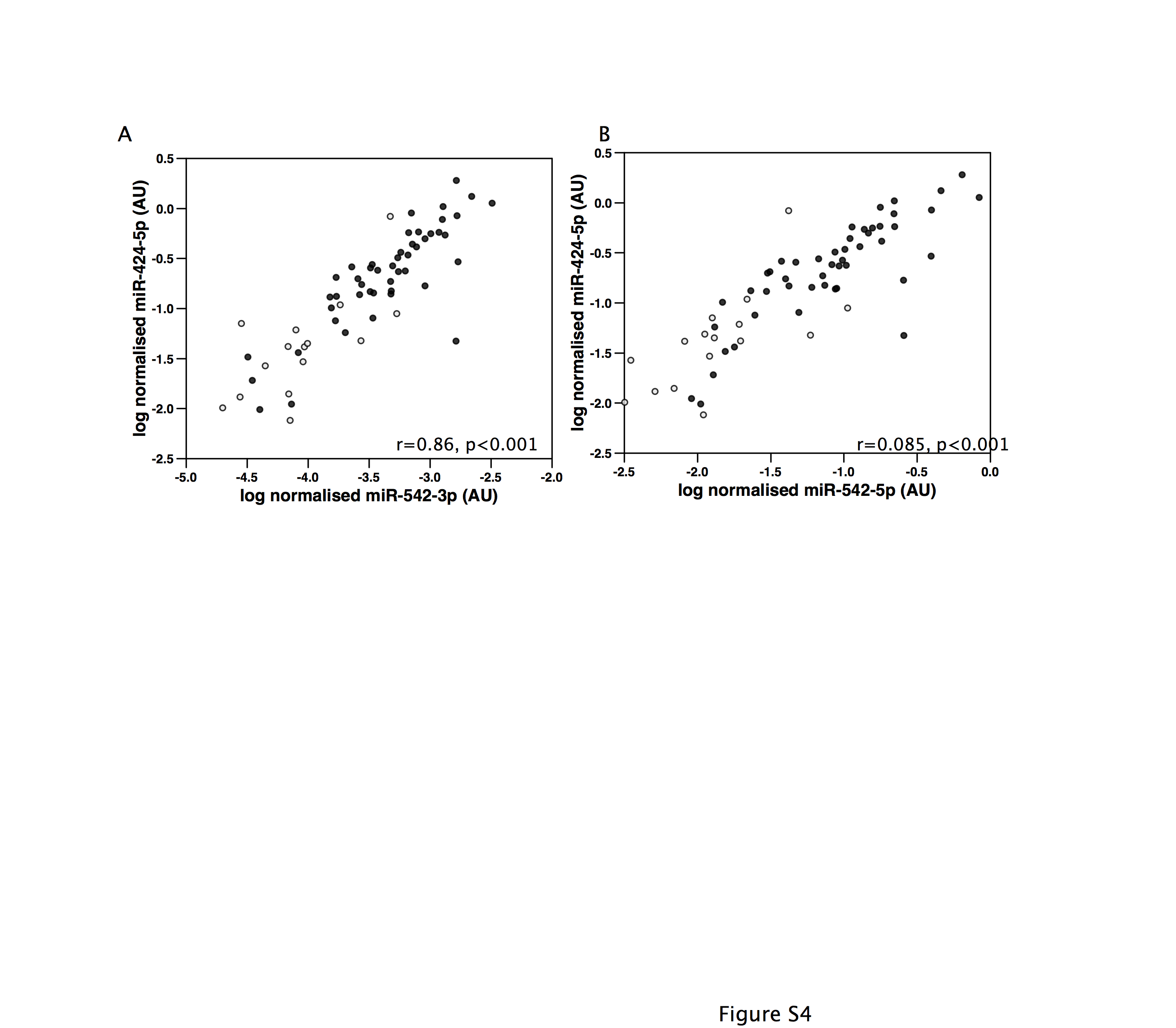

Supplement: Supplementary file 1 — Figure S1: Quantification of UBTF levels in transfected cells Figure S2: Puromycin quantification by Western blot Figure S3: EGFP and centralised nuclei in the electroporated mouse muscle Figure S4: miR‐424‐5p is associated with the expression of miR‐542‐5p and 3p in COPD muscle Figure S5: Quantification of normaliser genes Table S1: Primers used in this study Table S2: Physiological characteristics of the COPD cohort Table S3: Physiological characteristics of HSS cohort Table S4: Physiological characteristics of the ICUAW cohort Table S5: Physiological characteristics of aortic surgery patients Table S6: Selected Predicted gene targets of miR‐424‐5p [file JCSM-9-400-s001.zip › 424 and rRNA fig s4.tif]

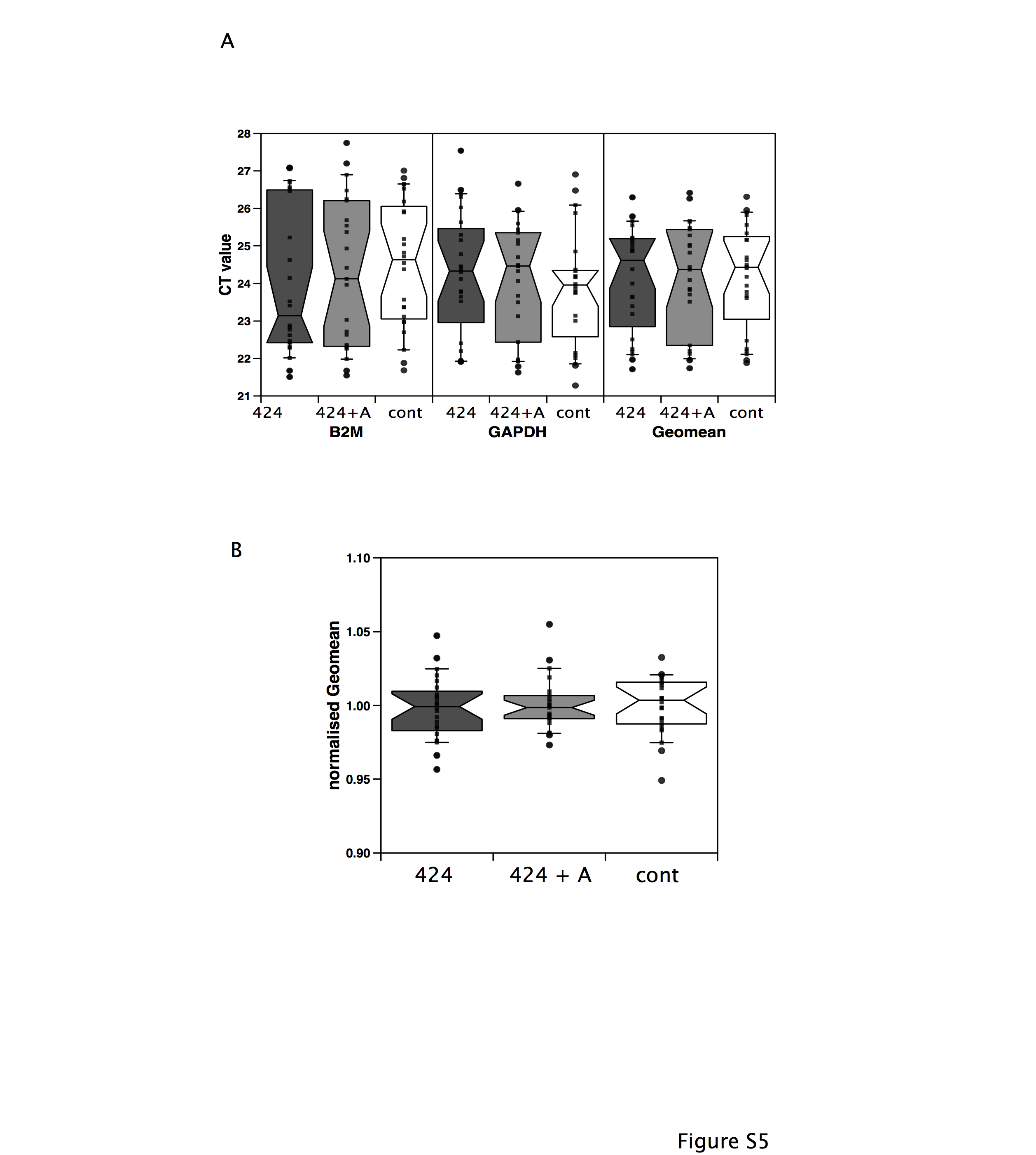

Supplement: Supplementary file 1 — Figure S1: Quantification of UBTF levels in transfected cells Figure S2: Puromycin quantification by Western blot Figure S3: EGFP and centralised nuclei in the electroporated mouse muscle Figure S4: miR‐424‐5p is associated with the expression of miR‐542‐5p and 3p in COPD muscle Figure S5: Quantification of normaliser genes Table S1: Primers used in this study Table S2: Physiological characteristics of the COPD cohort Table S3: Physiological characteristics of HSS cohort Table S4: Physiological characteristics of the ICUAW cohort Table S5: Physiological characteristics of aortic surgery patients Table S6: Selected Predicted gene targets of miR‐424‐5p [file JCSM-9-400-s001.zip › 424 and rRNA fig s5.tif]
